# Combining energy-based focal ablation and immune checkpoint inhibitors: preclinical research and clinical trials

**DOI:** 10.3389/fonc.2023.1153066

**Published:** 2023-05-01

**Authors:** Minhan Jiang, Steven Fiering, Qi Shao

**Affiliations:** ^1^ Department of Biomedical Engineering, University of Minnesota, Minneapolis, MN, United States; ^2^ Department of Microbiology and Immunology, Geisel School of Medicine, Dartmouth College, Hanover, NH, United States; ^3^ Dartmouth Cancer Center, Dartmouth Geisel School of Medicine and Dartmouth Health, Lebanon, NH, United States; ^4^ Department of Radiology, University of Minnesota, Minneapolis, MN, United States

**Keywords:** cancer, focal therapy, tumor ablation, immunotherapy, immune checkpoint, immunomodulation

## Abstract

Energy-based focal therapy (FT) uses targeted, minimally invasive procedures to destroy tumors while preserving normal tissue and function. There is strong emerging interest in understanding how systemic immunity against the tumor can occur with cancer immunotherapy, most notably immune checkpoint inhibitors (ICI). The motivation for combining FT and ICI in cancer management relies on the synergy between the two different therapies: FT complements ICI by reducing tumor burden, increasing objective response rate, and reducing side effects of ICI; ICI supplements FT by reducing local recurrence, controlling distal metastases, and providing long-term protection. This combinatorial strategy has shown promising results in preclinical study (since 2004) and the clinical trials (since 2011). Understanding the synergy calls for understanding the physics and biology behind the two different therapies with distinctive mechanisms of action. In this review, we introduce different types of energy-based FT by covering the biophysics of tissue-energy interaction and present the immunomodulatory properties of FT. We discuss the basis of cancer immunotherapy with the emphasis on ICI. We examine the approaches researchers have been using and the results from both preclinical models and clinical trials from our exhaustive literature research. Finally, the challenges of the combinatory strategy and opportunities of future research is discussed extensively.

## 1 Introduction

### 1.1 Overview of cancer treatments

Cancer is the second leading cause of death in the United States (1). Over the past century, numerous cancer treatments have been developed to improve patients’ survival and quality of life. According to NCI and ACS, there are a number of categories (2, 3) of cancer treatments that can be divided into several types, including surgery, radiation therapy, chemotherapy, immunotherapy, targeted therapy, and combination treatments. The U.S. Food and Drug Administration (FDA) has approved standards of care (SOC) to treat particular diseases depending on the type of cancer, the stage of cancer, and the patient’s disease history. SOC refers to a treatment that is accepted by medical experts as a proper treatment for a certain type of disease and that is widely used by healthcare professionals (4).

Surgical resection, chemotherapy, and radiotherapy are the most common SOC choices currently. Surgical resection has remained the first choice for most solid tumor cases. With advanced-stage cancer, chemotherapy, radiation, or both are usually suggested to control symptoms or to reduce the chance of local tumor recurrence and metastasis (5). However, these treatments come with limitations. Not all tumors are eligible for resection due to size, tumor location, or disease stage (6). When chemotherapy and radiation are used instead, the remaining cancerous cells may develop treatment resistance, resulting in failure to treat metastatic diseases in many cases (7). Another serious issue associated with chemotherapy and radiotherapy are side effects due to systemic toxicities and/or local damage to healthy tissues, which can range from hair loss and blood clotting problems to long-term organ damage (8, 9). Therefore, there is a desire for less invasive and more specific treatments that destroy the diseased tissue but have fewer adverse events and shorter recovery times, which has led to treatments such as focal therapies.

Energy-based focal therapy (FT), sometimes referred to as energy-based tumor ablation, is a rapidly growing field in loco-regional therapy (LRT) and interventional oncology (IO) for cancer management. FT uses targeted, minimally invasive procedures, usually performed with the help of image guidance, to treat and/or relieve the symptoms of cancer. It has been used for decades to treat solid tumors by effectively destroying tumors while preserving normal tissue and function so as to reduce side effects and cause minimal pain (10, 11). In addition, focal therapy can be used on unresectable disease, for instance, if the tumor is too large to be safely resected, or if it is intertwined with blood vessels and other vital structures, making safe removal impossible (12).

### 1.2 FT opportunities in the cancer immunotherapy revolution

The utility of leveraging the immune system to fight tumors has now been robustly validated. Cancer immunotherapy, most notably immune checkpoint inhibitors (ICIs), have definitively established that cancer can be treated very effectively by the immune system without directly eliminating cancer cells (13). ICIs are antibodies that disable molecular immune controls, i.e., checkpoints, that “turn off” antitumor immunity. There is strong interest in understanding the tumor immune microenvironment and how it impacts the response to immunotherapy (14).

While multiple strategies to enhance the response of ICIs have been proposed and studied, FTs have a unique set of advantages for combining with ICIs. Both the applied energy and the cell death caused by the applied energy causes perturbation of the tumor immune microenvironment. The unique ways FT modulate the immune system, especially compared to conventional cancer treatments, have started to be appreciated: FT has long been hypothesized to possess immunomodulatory properties as debulking removes the immunosuppressive tumor burden and consequently enables immune activation (15). By removing a tumor, surgery also eliminates the source of the immunosuppression, but it does not further support antitumor immunity. Unlike surgical resection, FT leaves tumor debris *in situ* and therefore creates antigens to enhance a locoregional and systemic antitumor immune response (16). Another relevant concept is that FTs support anti-tumor immune responses because of the cell death mechanisms induced by FTs. Unlike chemotherapy and radiation, FT induces cell death by extensive necrosis (16). Necrosis releases intracellular contents containing both the antigen and stimulating signals (among other danger signals) that activate T cells for the adaptive immune response to the FT-treated tumor.

This raises the significant possibility that FT can lead to *in-situ* tumor vaccination that generates local and systemic antitumor immunity while ICI helps effector T cells overcome immunosuppression in metastatic tumors not exposed to FT. Some recent work suggests that long-lasting systemic immunity against the tumor can occur after FT and ICI combined therapy (17). Therefore, it is crucial to understand the mechanism of action by which FT and ICI modulate the immune system, which is the topic of this review.

### 1.3 The synergy between FT and ICI

The motivation for combining FT and ICI in cancer management is based on the synergy between the two different therapies with distinctive mechanisms of action, as summarized in **Table 1**.

**Table 1:** The motivation of combinatory strategy relies on the synergy between focal therapy (FT) and immune checkpoint inhibitor (ICI).

FT helps ICI	ICI helps FT
* Reduce tumor burden * Increase objective response rate * Reduce side effects	* Reduce local recurrence * Control distal metastases * Provide long-term protection

In addition to tissue destruction, the immunomodulatory properties of FT have been documented. In recent decades, these immunomodulatory properties have been actively exploited to control cancer after the initial physical destruction (10, 18). FT improving ICI efficacy stems from the theory that FT expands the pool of tumor-specific CD8 effector T cells and reduce tumor immunosuppression, so there is more tumor-specific effector T cells to respond to ICI monotherapy. FT can also reduce ICI treatment-related side effects by improving the efficacy of ICI without increasing dosage.

On the other hand, despite the observation of abscopal effect and immunomodulation following various forms of FT (19–21), the immune response that follows FT alone is usually too modest to cause the system-wide, sustained anti-tumor effect needed to destroy distant metastases. The addition of systemic immunomodulation provided by ICI in the combination treatment has potential for inducing effective long-lasting anti-tumor immunity to combat both local recurrence and metastasis.

This combinatorial strategy has shown promising results in a variety of studies, especially since 2004 (22) and 2011 (23), when the first preclinical study and the first clinical trials were reported, respectively. Since then, more forms of FT and various types of ICI has been evaluated in a wide range of cancer types. More than half of the published preclinical studies and newly launched clinical trials took place within 5 years of the writing of this review. Besides appreciating the explosion of research and the enormous opportunities of the combinatorial strategy of FT and ICI, understanding the synergy also calls for understanding the physics and biology behind this emerging area.

In this review, we start by introducing different types of energy-based focal therapy. In Section 2, we cover the biophysics of FT based on heating and hyperthermia, freezing and electric membrane disruption, and how each utilization of energy leads to focal tissue destruction. In addition, practical and immunomodulatory advantage of energy-based FT compared to conventional cancer therapy is discussed. In Section 3, we present important aspects of immune responses following FT due to its strong association with immunogenic cell death (ICD), as understanding the immunomodulatory effects of FT serves as the foundation of designing and optimizing combinatory approaches with cancer immunotherapy. We continue by introducing cancer immunotherapy (Section 4) with an emphasis on ICI (Section 5) to provide the basis for the synergy between FT and ICI. Later, we examine the approaches and outcomes researchers have been using and the results from both *in vivo* models (Section 6) and clinical trials (Section 7) from our exhaustive literature research. Finally, the challenges of the combinatory strategy (Section 8) and opportunities of future research (Section 9) are discussed extensively.

## 2 Energy-based focal therapy (FT) and its clinical applications

Energy-based FT, or focal tissue ablation, is a form of locoregional therapy that relies on energy to destroy tissues without affecting the rest of the body. Cancer focal therapies can be categorized into thermal and nonthermal techniques, depending on the form of energy leading to tissue destruction. Thermal techniques can be high-temperature-based, such as radiofrequency ablation (RFA), microwave ablation (MWA), high-intensity focused ultrasound (HIFU), laser ablation and nanoparticle (NP)-mediated hyperthermia, or low-temperature-based such as cryoablation. Nonthermal techniques include irreversible electroporation (IRE) and histotripsy. Ablation techniques, the source of energy, and their clinical targets are summarized in **Table 2**.

**Table 2:** Overview of energy-based focal ablative techniques.

Energy	Ablation technique	Energy source or modality	Common clinical target (24–31)
Heating and hyperthermia	RFA	Oscillating electrical current	Bone, liver, lung, kidney, and prostate
	MWA	Oscillating electromagnetic field	Liver, lung, kidney, and bone
	HIFU	Ultrasound	Prostate, bone
	LITT	Laser	Liver, bladder, brain, and prostate
	NP-mediated hyperthermia	NIR light or alternating magnetic field	—
Freezing	Cryoablation	Cryogen or Joule-Thomson effect	Prostate, kidney, liver, breast, skin, lung, and bone
Electric membrane disruption	IRE	Electrical pulses	Liver, prostate and pancreas
Mechanical destruction	M-HIFU, histotripsy	Ultrasound	—

RFA, radiofrequency ablation; MWA, microwave ablation; HIFU, high-intensity focused ultrasound; LITT, laser-induced interstitial thermotherapy; NP, nanoparticle; IRE, irreversible electroporation; NIR, near-infrared; M-HIFU, mechanical high-intensity focused ultrasound. The list consists of the energy source, its corresponding focal ablation technique, and its common clinical targets (FDA approved and covered by insurance, clinical trials not included) for cancer therapy. -, used in preclinical study or clinical trial.

It should be noted this review focuses on physical energy as the direct and immediate cause of cell death, and we do not cover every form of locoregional therapy. While widely used in clinical settings, some locoregional therapies rely on cell death mechanisms other than energy-cell interaction. Techniques not discussed in this review include but are not limited to (1) ionizing radiation therapy (32), such as external-beam radiation therapy, stereotactic body radiation therapy, selective internal radiation therapy, high dose rate brachytherapy, and transarterial radioembolization; (2) chemical ablation (33), which relies on focal cellular toxicity and protein denaturation to produce cellular necrosis, typically by focal injection of chemical ablation agents such as ethanol, acetic acid, and hypertonic sodium chloride solution; and approaches sometimes called thermochemical ablation and electrolytic ablation, including (3) embolization (34), such as transcatheter arterial chemoembolization and portal vein embolization; (4) local cytotoxic therapy, such as photodynamic therapy (35) and cold atmospheric plasma (36); (5) local chemotherapy, such as electrochemotherapy (37) and local administration of cytotoxic agents (e.g., doxorubicin, benzalkonium chloride, silver nitrate, etc.) (38); and (6) others such as gene electrotransfer (39, 40).

### 2.1 Heating and hyperthermic focal therapy

Heat has long been studied as a crucial mechanism for cellular damage; different temperature ranges can lead to distinct biological mechanisms. As such, many different energy-based focal ablative techniques exist, as shown in **Table 2**. In all of these cases, raising the temperature can lead to cell injury and eventually death. For example, as the temperature is raised to 39°C, blood perfusion in tissues will increase to protect tissue, but sustained heating above 42°C can lead to vascular stasis and thrombosis followed by ischemic necrosis and death. At the cell level, temperatures between 41°C and 45°C led to diminished or halted metabolism and impaired DNA repair. Extensive reviews on the subject of hyperthermic cellular and tissue destruction are available (41, 42).

Radiofrequency ablation (RFA) occurs by applying an oscillating electric current (typically between 450 and 500 kHz) to the target tissue through direct placement of one or more interstitial electrodes into the tumor. Tissues further away from the electrode are heated primarily by thermal conduction (43, 44). The delivery of RF energy and resulting lesion size are determined by the tissue electrical conductivity and thermal conduction. During RFA, water vapor, desiccation, and charring can occur, which increases the electrical impedance significantly (43, 45). As a result, RF ablation zones can vary widely according to electrode design (e.g., size, shape, or even internal cooling), electrical control (i.e., voltage, current, and power), local tissue environment (i.e., thermal conduction and blood flow). Different RF probe designs (such as internally cooled wet electrodes) can address desiccation and even decrease electrical impedance (46).

Microwave ablation (MWA) relies on the electromagnetic field generated by an intratumorally placed antenna to generate dielectric heating. The most common frequencies used for microwave ablation are 915 MHz, 2.45 GHz, and broadband frequencies between 1 GHz and 10 GHz. Polar molecules (primarily water) within the tissue continuously realign with the oscillating electromagnetic field, effectively increasing kinetic energy. The ability to cause hyperthermic injury is determined by device design (antenna design, number, and orientation), MW characteristic (power and frequency), tissue electrical properties (dielectric constant, electrical conductivity, and relative permittivity), and tissue thermal properties (43, 47).

High intensity focused ultrasound (HIFU) uses multiple high-intensity non-ionizing ultrasound beams and focuses them on a selected focal area to destroy the target tissue. HIFU systems typically operate in the frequency range of approximately 500 kHz and 7.5 MHz. HIFU causes tissue injury through two primary mechanisms: thermal damage due to absorption of the applied acoustic energy and mechanical damage due to acoustic cavitation. The amount of acoustic energy transferred from the acoustic wave to the tissue is directly proportional to the intensity of the wave and the innate absorption coefficient of the targeted tissue (47). (Micro) bubble can be injected or created during HIFU or tissue boiling. The presence of bubble can enhance heating effect owing to their ability to generate higher harmonics of the exciting frequency and lower the cavitation threshold, allowing lower energies to be used for ablation (48).

Laser ablation, which refers to laser-induced interstitial thermotherapy (LITT) in our context, also known as stereotactic laser ablation (SLA), is performed by implanting a laser catheter into the tumor. It uses high-intensity lasers to generate heat. Heat is generated by optical absorption and is then conducted to the rest of the tissue to shrink or destroy tumors (27). Temperature within the lesion is usually measured throughout the procedure using MRI thermometry. Laser penetration into the tissue is affected by the specific optical properties of the tissue. The effect of laser ablation is influenced by a number of factors: laser light wavelength, laser settings (laser power, laser energy, and treatment time), physical properties of the tissue, and the emission characteristics of the optical applicator (49).

NP-mediated hyperthermia generates heat based on the unique and highly tunable optical or magnetic properties of nanomaterials. Based on the energy source, NP-mediated hyperthermia can be categorized as photothermal therapy (PTT) or magnetic hyperthermia therapy (MHT), also known as magnetic fluid hyperthermia (MFH). PTT involves the application of normally benign light wavelengths (most often NIR light) in combination with efficient photothermal agents (e.g., gold or carbon nanomaterials) that convert the absorbed light to heat (50). MHT relies on magnetic nanoparticles to transform electromagnetic energy from an alternating magnetic field (AMF) to heat (51, 52). In practice, MHT has been studied on pre-clinical models, and has also been used in clinical trials to treat deep-seated, terminal, unresectable tumors, such as glioblastoma multiforme or prostate tumors (53). Due to the limitations of laser light penetration, PTT has been studied in preclinical models and several clinical trials for more superficial cancers, such as breast cancer, melanoma, lung cancer, and colorectal cancer (54).

### 2.2 Cryogenic focal therapy

Cryoablation relies on cryogenic temperature to cause cold injury and destroy target tissue, as also featured in **Table 2**. The freezing process results in intracellular and extracellular ice formation, dehydration, and vascular injury. The mechanism of tissue injury varies with the freezing rate and final tissue temperature, as well as tissue susceptibility. A slow rate of freezing favors the formation of extracellular ice crystals, which leads to a hypertonic extracellular environment and osmotic cell shrinkage from fluid shifting out of the cell, increasing cell dehydration and death. Fast freezing to lower temperatures promotes the formation of intracellular ice crystals, which results in direct cell injury because of damage to the cell membrane and organelles (55, 56). Cold-induced vascular injury usually leads to platelet aggregation, microthrombosis, and ischemia, causing further coagulative necrosis. Apoptosis can also occur in some cases in the peripheral zone of sublethal cold temperatures and contributes to tissue damage (55, 56).

Cryoablation is performed using needle-like probes, where rapid cooling is achieved by circulating cryogen (e.g., liquid nitrogen) or by the Joule-Thomson effect (45). Heat transfer in surrounding tissue is governed by passive thermal diffusion (43). During the application of cryoablation, an ice ball is formed, allowing for precise monitoring of the ablation zone by ultrasound, CT, or magnetic resonance imaging. However, the temperature that is necessary for cell lethality is between -20°C and -40°C, which means the lethal isotherm lies inside of the visualized ice ball, making it difficult to destroy tumor completely while not damaging surrounding healthy tissue (57).

### 2.3 Irreversible electroporation (IRE)

IRE, as also featured in **Table 2**, applies pulses of electrical fields (thousands of V/cm) that last from nanoseconds to milliseconds to create permanent and lethal nanopores in the cell membrane that disrupt cellular homeostasis and induce cell death (58, 59). IRE relies on the flow of current through tissue to induce cell death; therefore, electrical conductivity is the most important factor determining the distribution of the electric field. In addition, pulse parameters are thought to influence the profile of cell death within targeted tissue and therapeutic outcomes (58).

Unlike thermal ablation techniques, IRE is not susceptible to heat sink effect, which occurs when heat or cold is absorbed by flowing blood or air and carried away from the area of ablation, thereby limiting the effectiveness of ablation when the target lesion is in close proximity to a large blood vessel (45). In addition, IRE can preserve major vascular and ductal structures in the ablated region, offering the benefits of short treatment time and reduced collateral thermal injury (58). These unique advantages make IRE a good option for treating tumors in specific locations (e.g., pancreas).

### 2.4 Mechanical tissue disruption

Mechanical tissue disruption/destruction, also known as mechanical HIFU ablation, is achieved by exposing tissue to repeated short- (microsecond to millisecond) duration pulses of high-intensity ultrasound with low duty cycles (60). The tissue is fractionated in a controlled manner, compared to thermal destruction by HIFU. There are different methods of mechanical tissue disruption using focused ultrasound with distinctive bio-effects, such as low-intensity (< 1 kw/cm^2^) focused ultrasound techniques [sometimes refer to ultrasound irradiation (61)], high intensity (1-10 kw/cm^2^) techniques that increases cell permeability and extremely high-intensity (>10 kw/cm^2^) focused techniques [for example histotripsy (62), boiling histotripsy (63)] leading to tissue fragmentation. In addition, the collapse of bubbles or gas-filled cavities create an extremely large pressure shock wave capable of inducing fragmentation of tissue into subcellular levels (64).

### 2.5 Novel energy-based focal therapies

There are numerous energy-based cancer therapies being developed and tested. Some techniques have gained FDA approval, such as tumor treating fields, which rely on mild electrical fields for tumors including glioblastoma multiforme (GBM) (65) to interrupt the cancer cells’ ability to divide. Some of these therapies are in clinical trials, such as cryo-thermal therapy, which combines cooling by LN_2_ and heating by RFA. Some are still in developmental stages, for example cryoelectrolysis, which combines electrolysis and freezing. However, due to limited knowledge available, their immunological effect and/or their combinatory approach with immunotherapy are not covered in this review.

### 2.6 Advantages and drawbacks of FT

Focal therapy (FT) has been proposed as a minimally invasive option for treating localized disease with the aim of minimizing the side effects associated with radical treatment while maintaining the oncological benefit of local treatments (66). FT is usually recommended when other common treatments are not appropriate due to tumor size, tumor location, or disease stage. It is useful in relieving pain and slowing disease progression and is often combined with other treatments (as an adjuvant), such as hormone therapy, chemotherapy, radiation therapy, or surgery. The recovery time for FT is typically shorter than for surgery or radiation therapy. In fact, it usually does not require an overnight hospital stay (67, 68). FT can also be used as a salvage or palliative therapy, when other commonly used treatments (e.g., radiation therapy or surgery) fail, or patients cannot tolerate these treatments (69).

FT offers a range of practical advantages over conventional surgical resection, radiation therapy and chemotherapy. Being a cost-effective alternative to surgery, minimally invasive FTs are indicated for a large range of malignancies at an early stage or for those not eligible for surgery. They have a better complication/risk profile than radical surgery and can be used in patients who are not fit enough for or decline radical options (18). When the tumor is not suitable for resection and has limited response to chemo/radiation due to high tumor burden, prior radiation treatment of the site, or drug resistance, an ablation protocol can be a good treatment plan in order to significantly decrease the primary tumor burden and develop systemic anti-cancer immunity. Unlike chemotherapy or radiation therapy, FTs rely on physical energy for the destruction of cancer cells and therefore circumvent the resistance of tumors that received prior treatments, making FTs a great option for repeated treatment (70).

Although focal therapy can offer several advantages compared to traditional treatment, clinical use of FT as a first-line treatment is quite limited. In the clinic, cryosurgery is a first-line treatment in dermatological disorders for early-stage skin cancer, retinoblastoma, and precancerous growths on the skin and cervix (71). Thermal ablation and IRE are not commonly available. The biggest problem preventing FT from being considered SOC is that a large percentage of cancers, including melanoma (72), prostate (73), liver (74), pancreas (75), and kidney (74), treated by FTs still go on to fail by local and systemic recurrence. Even a small amount of residual tumor after focal therapy can lead to treatment failure. Furthermore, long-term safety and efficacy data for FT for various cancers is limited, largely due to the lack of randomized controlled trials comparing specific FTs to SOC therapies in assessing both oncologic and functional outcomes (76).

## 3 Immunological effects of focal therapy

**Figure 1:**
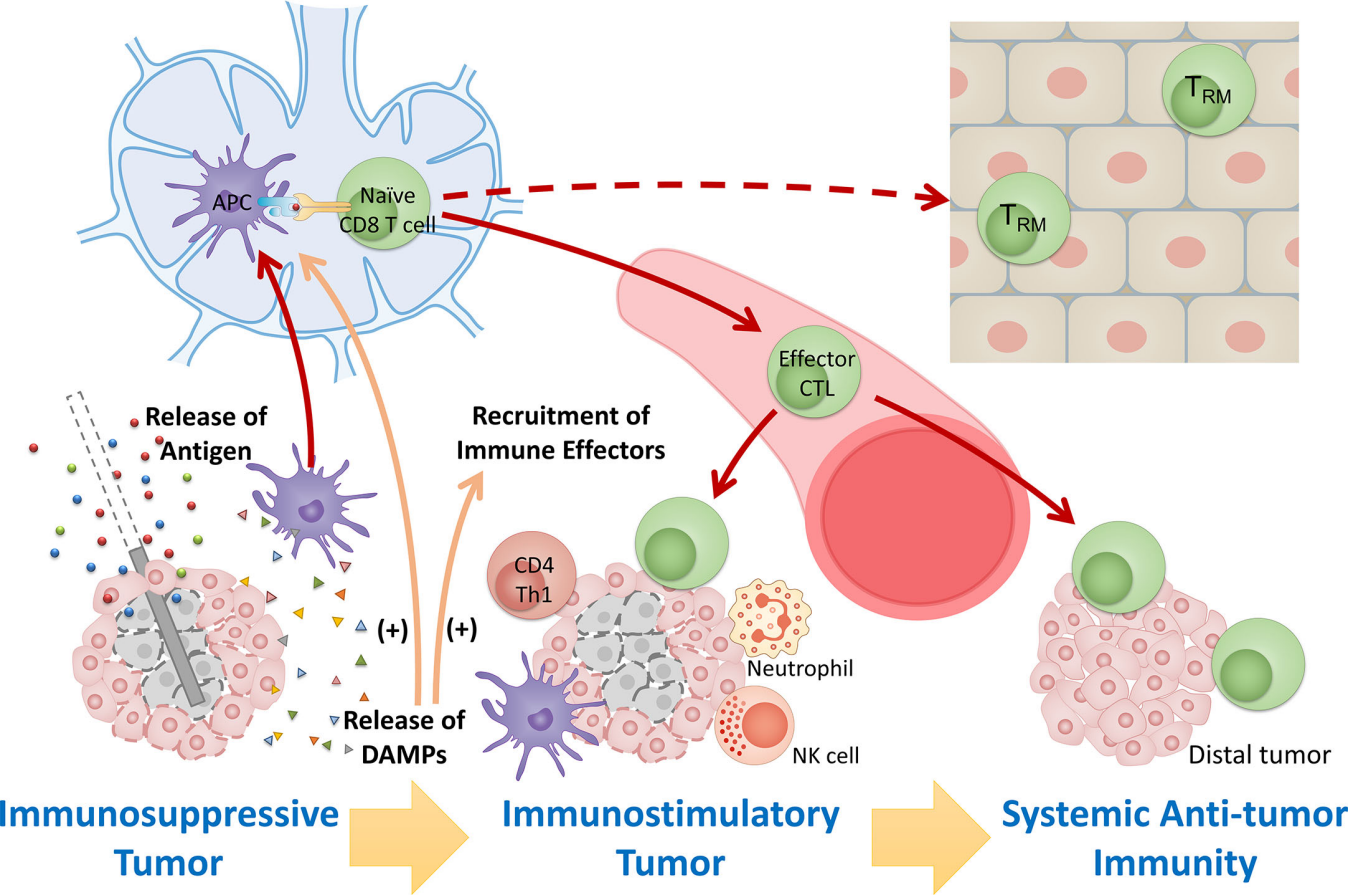
Overview of immunomodulatory effect after FT. Tumor destruction by FT releases large amount of tumor antigens and immunostimulatory DAMPs, leading to antigen presentation and activation of immunity, recruitment of immune effectors, and mitigation of immunosuppression. The response stimulates systemic anti-tumor immunity and induces maintenance of tissue-resident memory cells involved in local immunosurveillance, leading to potential long-lasting protection against cancer.

There is a complex and dynamic interplay between the immune system and cancer. The immune system plays a central role in the balance of cancer progression and cancer suppression. Cancer manifests a variety of mechanisms that manipulate the immune system to support the cancer and suppress antitumor immunity. Adding further complexity, the treatment of cancer can also profoundly modulate the immune system, which has been studied extensively in *in vivo* models and in clinical studies (77). In short, there are both molecular (**Table 3**) and cellular (**Table 4**) modulators of the immune response, as depicted in **Figure 1**, which we will explore further below.

**Table 3:** Cytokines and other small molecules observed following focal therapy and their immunomodulatory effect.

Name	Examples of effect on immune cells following Focal Therapy
Cytokines (& chemokines)	
IL-1	Promote Th1 response, promote the activation of DCs and CTLs (78)
IL-6	Support T cell proliferation and survival, support T cell trafficking to the tumor, interfere the development and maturation of DCs, increase MDSCs, inhibit Th1 response (78)
IL-8	Recruit neutrophils and MDSCs into the tumor microenvironment (78, 79)
IL-12	Stimulate the growth and function of T cells, enhance the cytotoxic activity of NK cells and CD8+ cytotoxic T lymphocytes (80)
IL-18	Facilitate Th1 response, induce IFN-γ production with IL-12, involved in T cell differentiation (78)
TNF-α	Regulate T cell differentiation and function (78)
Type I IFN	Stimulate both macrophages and NK cells to elicit an anti-tumor response (81, 82)
IFN-γ	Promote NK cell and CTL activity, increase antigen presentation, can become pro-tumorigenic through chronic inflammation (78)
IL-10	Suppress the secretion of pro-inflammatory cytokines, dampen antigen presentation and T cell activation; help with helper T cell functions and T cell immune surveillance (83)
TGF-β	Inhibit B cell proliferation and the maturation of macrophages, promote the differentiation of Treg cells, downregulate cytokine production (83)
Other molecules	
HSP70	Support uptake and processing of tumor antigens by DCs, enhance MHC class I expression tumor cell surface, enhance susceptibility of tumor cells by CTLs (84, 85)
HMGB1	Induce migration and maturation of immune cells, release cytokines and other inflammatory mediators (86)
ATP	Attract DC and phagocytes, stimulate the priming of IFN-γ-producing tumor antigen-specific CD8+ T cells (87)
Calreticulin	Promote phagocytic uptake of cancer cells by immune system (87)
Degradation products (e.g., DNA, RNA, uric acid)	Induce DC maturation and activation, mediate innate immune response and promote inflammatory response (87, 88)

**Table 4:** Major immune cells involved in FT-induced anti-cancer immune response, based on their respective functional roles.

Cell type	Function in anti-cancer immunity (89–96)
DC	* Capture and process antigen, display antigen on the cell surface bound to MHC-I or MHC-II * Present antigen to naive T cells and activate T cells; cross-present to activate CD8+ T cells
Cytotoxic CD8+ T cells	* Recognize tumor cells by the antigen peptide on MHC-I expressed on cancer cells * Kill cancer cells *via* release of granules or induction of FasL-mediated apoptosis
Helper CD4+ T cells	* Provide help (stimulus) for priming of CD8+ T cells, help activation and licensing of DCs for induction of DC maturity, help recruit immune cells (e.g., NKs, M1 macrophages) * Help B cells produce antibodies to induce humoral responses against tumor antigens
Cytotoxic CD4+ T cells	* Direct anti-tumor activity through effector cytokines (IFN-γ and TNF-α) secretion or direct cytotoxicity
CD4+ Treg cells	* Suppress anti-tumor immune effector responses in the TME, primarily by promoting an immunosuppressive microenvironment
NK cells	* Cytotoxic NK cells kill cancer cells by releasing cytotoxic granules, through antibody-dependent cellular cytotoxicity mechanism, or *via* FasL-mediated pathway * Regulatory NK cells produce pro-inflammatory cytokines, such as IFN-γ
B cells	* Regulatory B cells support carcinogenesis, tumor progression and metastasis * Tertiary lymphoid structures (TLSs) B cells coordinate anti-tumor immune responses through multiple mechanisms (e.g., antigen presentation, cytokine secretion, tumor-specific antibodies secretion)
Macrophages	* M1 macrophages have a proinflammatory phenotype with pathogen-killing abilities, production of proinflammatory cytokines and higher antigen-presenting capacities * M2 macrophages have an anti-inflammatory phenotype with higher phagocytic activity, and promote tissue remodeling, neo angiogenesis, and tumor progression
Neutrophils	* N1 cells display proinflammatory and anti-tumor functions * N2 cells display immune suppressive protumorigenic activity
MDSC	* Suppress CD4+ and CD8+ T cell activation and function, drive and recruit Tregs * Inhibit innate immunity * Promote tumor progression
Memory T cells	* Play a crucial role against tumor recurrence *via* direct cytotoxic activity and immune-stimulating functions

Immune cells can be either immune-promoting and anti-cancer, or immune-suppressive and protumorigenic, depending on their phenotypes.

Cancer focal ablations dramatically change the physical tumor environment and function through the direct or indirect induction of cell death. Each type of ablation causes a distinct type of cell stress and tissue destruction with variable immunological outcomes (16). Focal ablation is set apart from conventional treatment methods (e.g., chemotherapy, surgery) due to its strong association with immunogenic cell death (ICD) and because it is characterized by the tissue injury response and wound healing processes (16, 55, 97). Therefore, FT can lead to a strong anti-tumor immunity by direct stimulation and indirect support involving both adaptive and innate immune response (10). In order to understand how focal therapy impacts the anti-cancer response at the primary tumor and, furthermore, how it can generate an effective abscopal effect and long-term immune protection, the unique features of these complex and variable interactions must be explored.

ICD is cell death characterized by molecular signals that activate innate immune cells, including antigen-presenting cells and this leads to tumor antigen presentation to T cells, activating the adaptive immune system against cancer (87, 98). ICD is mainly mediated by release of damage-associated molecular patterns (DAMPs) (87) and can strongly support the immune system in support of cancer therapy (98). It is worth noting that cancer cell death can be immunogenic or nonimmunogenic and that cell death in tumors without any intervention is common, often due to hypoxia. This type of cell death is not immunostimulatory because of the lack of DAMPs.

In addition to ICD, the immunological effects of focal therapy following acute physical destruction of a tumor are strongly influenced by the details of cell death, tissue injury, inflammation induction, and subsequent wound healing processes. Upon tissue injury, neutrophils and macrophages present in local tissue become activated, and the mast cells release cytokines and vasoactive substances. This results in the recruitment of white blood cells and the initiation of the wound healing process (99). Wound healing is typically divided into four phases: blood clotting (hemostasis), inflammation, tissue growth (cell proliferation), and tissue remodeling (maturation and cell differentiation) (100). Among these stages, inflammation is considered an important reaction after FT and may determine the success or failure of treatment. Inflammation is accompanied by the release of inflammatory mediators, vasodilation, and the migration of leukocytes, mainly neutrophils and macrophages, into the tissue (101). In addition to these various interactions with the immune system, ongoing research is also examining different pathways focal therapies modulate.

Substantial preclinical and clinical studies using different cancer models to investigate how specific focal therapies modulate antitumor immunity have demonstrated that tumor destruction by FT releases large amounts of tumor antigens and immuno-activatory DAMPs, leading to antigen presentation, activation of immunity, and recruitment of immune effectors (10, 16). These immune responses after FT potentially “reset” the tumor microenvironment from an immunosuppressive state that largely excludes invading immune cells to an immunostimulatory state that is more susceptible to local control by the immune system, further stimulating systemic anti-tumor immunity and inducing maintenance of memory cells involved in local immunosurveillance (**Figure 1**).

In this review, the short-term and long-term immune response initiated by cancer focal therapy are categorized into 6 aspects: (1) exposure of tumor antigens, (2) ICD and release of DAMPs, (3) antigen presentation and activation of immunity, (4) recruitment of immune effectors, (5) modulation of immunosuppressive cell types and (6) maintenance of memory cells. Here we describe the basis of each pathway and the modulations that focal therapies have in common. The DAMPs, cytokines, and chemokines that have been observed following FT together with their primary roles in the immune response against cancer are summarized in **Table 3**. Major immune cells involved in FT-induced anti-cancer immune response and their functions are summarized in **Table 4**.

### 3.1 Tumor antigens

Tumor antigens are proteins or carbohydrates that are recognizable by T cells or B cells as antigens and against which an antitumor immune response can be generated (102, 103). Based on expression profiles, there are two general classes of tumor antigens: tumor-specific antigens (TSAs), which are expressed only by cancer cells, and tumor-associated antigens (TAAs), which represent the mutated counterparts of proteins expressed by normal tissues and can often be expressed by many patient tumors of a given type (103). Most tumor-specific antigens are due to mutations that accumulate in cancer cells and are often termed neoantigens, which are generally specific for a given patients tumors. The expression of cancer antigens reveals the accumulation of a variable number of genetic alterations and the loss of normal cellular regulatory processes, distinguishing cancer cells from normal cells (104). These differences are fundamental for cancer cell recognition and clearance by the adaptive immune system (102).

During tumor focal destruction, when cancer cells are going through mostly necrosis, cellular contents containing tumor antigens are exposed to the extracellular space due to the loss of plasma membrane integrity, the disruption of cell organelles, and the degradation of nucleic acids and proteins (105, 106). Antigens that reside in plasma membrane blebs and apoptotic bodies can also be released after FT when the cells are undergoing apoptosis (106). These antigenic materials enter the local lymphoid drainage in the form of soluble proteins or aggregates (22). There is evidence that tumor-cell derived exosomes contain an enriched amount of tumor antigens (107) and the release of exosomes is increased when tumor cells experience stress, such as hypoxia (108) and heat (109). When antigens enter the bloodstream, they may bind to serum antibodies to form immune complexes, which have been shown to stimulate cross-presentation, CD8+ CTL responses, and cellular tumor immunity (110).

Even though antigens are released and/or exposed by all forms of focal therapy, the quantity and quality of released antigens are very different among focal therapy approaches, leading to variable immunogenicity. For example, cryoablation is considered to induce higher post-ablative immunogenicity compared to high-temperature-based methods (e.g., MWA and RFA) based on the assessment of the serum antigen level and the percentage of antigen-loaded DCs in the draining lymph node, and the general evaluation of inflammatory responses (111–113). A popular explanation is that thermal ablation tends to cause protein denaturation, therefore decreasing the amount of soluble protein accessible to the immune system, as well as reducing the antigenicity of protein (10, 111, 114, 115). Intense heat typically results in coagulation, further hindering the transport of antigenic materials. In contrast, cryogenic methods tend to preserve protein structure without significant denaturation (10, 114). In addition, IRE has been shown to release a substantial quantity of antigen to stimulate immune response (116). As an electrical-based ablation technique, IRE delivers high-voltage electrical pulses to the tumor to cause membrane rupture, induces severe leakage of intracellular contents with less drastic alterations in protein conformation compared to thermal-based methods, and presumably contributes to superior antigen release (58, 116). Furthermore, the preservation of extracellular structures, including lymphatics and blood vessels, after IRE treatment (58) can facilitate the distribution of antigens into circulation and later interaction with APC. However, it is important to avoid oversimplifying the difference in antigen accessibility by looking only at the categorization of FT therapies: comprehensive immunological studies are needed, and the details of focal therapy matter immensely.

### 3.2 Generation of DAMPs

DAMPs also known as danger-associated molecular patterns and danger signals, are host biomolecules released from or exposed on dying, stressed, or injured cells that act on pattern-recognition receptors to activate the innate and, subsequently, the acquired immune systems (16, 87). The generation of DAMPs is recognized as a prominent immunogenic characteristic of ICD, and DAMPs play a critical role in inflammatory responses (87, 88) and tissue healing after inflammation (88). Therefore, the role of DAMPs in cancer focal therapy immune stimulation is central.

During focal ablation, DAMPs are released from or exposed on the ablated tissue, including tumor cells, stromal cells, endothelial cells, and immune cells, as well as released due to the disruption of local extracellular matrix (117). These DAMPs then bind to phagocytosis receptors, purinergic receptors, and pattern-recognition receptors to initiate a series of reactions such as the local production of inflammatory cytokines (TNF, IL-1, IL-6, and IL-8) from innate immune cells (16, 87). Meanwhile, neutrophils, macrophages, dendritic cells, and other immune cells are recruited to the ablated site and are locally modulated by the DAMPs. The recruited cells will release cytokines and chemokines, which in turn coordinate with local cells to further modulate the immune response, ultimately leading to the activation of anti-cancer immunity (16). To some extent, the release of DAMPs, cytokines, and chemokines following FT could be used to rapidly evaluate the immunogenic effects of a treatment.

Cytokines are widely studied in the immunological response to focal ablation through evaluation of serum levels of cytokines *in vivo* and clinically. Heat shock proteins, ATP, HMGB1, calreticulin, and end-stage degradation products (such as DNA, RNA, and uric acid) have been largely limited to *in vitro* study.

Following thermal ablation, increase of pro-inflammatory cytokines (e.g., IL-1, IL-6, IL-8, IL-18, and TNF-α) are common for several hours to days (118–120), while anti-inflammatory cytokines (e.g., IL-10 and TGF-β) are also released. Cryoablation releases a similar category of cytokines, but in different quantity. For example, IL-1, IL-6, and NF-κB-dependent cytokines such as TNF-α are released in higher quantities after cryoablation than after RFA and MWA (114, 121). The large amount of pro-inflammatory mediators after cryoablation may induce a systemic inflammatory response syndrome (SIRS), leading to a phenomenon called cryoshock. Cryoshock can cause severe consequences, including disseminated intravascular coagulopathy, multi-system organ failure, or even death (122). SIRS does not occur when heat-based focal ablation techniques are used (121). After heat treatment, chemokines such as CCL2, CCL4, CCL5, and CCL10, which attract DC and T cells into the tumor, are detected (123). The release of ATP, HMGB1, and degradation products, as well as calreticulin externalization, are often detected after focal treatment is applied to cell suspension (105); however it is unclear how different ablation methods influence the amount of released molecules. Following IRE, ATP- HMGB1-mediated signaling are significantly up-regulated (124), suggesting that IRE might be an effective strategy to induce immunogenic cell death. The elevated expression of HSP 70 has been reported after both thermal ablation and cryoablation (125, 126), while the elevation in the thermal ablation group is much higher than with cryoablation (126). Furthermore, increased serum levels of HSP70 are correlated with better survival in patients treated with RFA (127).

### 3.3 Antigen presentation and activation of immunity

After the maturation and activation of APCs (particularly DCs) stimulated by DAMPs, dying tumor cells or released antigens are absorbed and processed by APCs, then the activated APCs travel to the draining lymph nodes, where antigens are presented to naive T cells (128). Cross-presentation and cross-priming are critically important for CD8-mediated immune response, in which MHC-I binds peptides derived from exogenous proteins internalized by endocytosis or phagocytosis and then primes naive CD8+ T cells (128). Following this interaction between peptide-MHC-I complexes and TCR, in the presence of costimulatory signals and/or cytokines, naive cytotoxic T cells become activated and proliferate. Following initial proliferation, they migrate into the circulatory system as effector T cells to identify cancer cells and ultimately eradicate tumors (128).

Antigen presentation and the activation of adaptive immunity rely on the accessibility of antigens and activation status of DCs and T cells, which are modulated by immunostimulatory signals such as DAMPs released after FT. This process is typically evaluated through the assessment of activation markers/phenotype change on DCs and T cells, as well as proliferation of T cells in lymph nodes and peripheral blood. Following cryoablation and thermal ablation in mouse models, a significant amount of antigen-positive DCs and an increase of DC activation markers (e.g., CD80/86) within the draining lymph nodes have been demonstrated (113, 129, 130). Increased tumor-specific T cell activation in regional lymph nodes and expanded CD8 T cell populations have also been widely detected after focal ablation (131). Clinically, the changes in T cell population in the peripheral blood of patients with different tumor types following thermal ablation or cryoablation have been evaluated. Peripheral blood CTL numbers and CTL/regulatory T cells (Treg) ratios increased significantly after thermal ablation but remained unchanged after cryoablation (132). In an *in vitro* study, IRE has shown superior induction of CTL response compared to cryoablation and heat-based treatment (116). Nevertheless, carefully controlled studies evaluating antigen presentation and activation of CTL will productively guide the choice and implementation of focal therapy to improve anti-tumor immune response.

There is a growing interest in the role of CD4+ T cells in anti-tumor immunity (133–135). Th1 type CD4+ cells most likely support a robust anti-tumor immune response, while CD4+ Treg cells are immunosuppressive (96). The influence of FT on CD4+ T cell differentiation and the role of CD4+ T cells in FT-induced immune response is being actively investigated (136, 137). For example, one study found that cryo-thermal therapy, but not RFA, led to a strong neoantigen-specific CD4+ T-cell response that mediated the resistance to tumor challenge (136).

In addition to cellular adaptive immunity, which is mediated by T lymphocytes, humoral immunity, which is an antibody-mediated response, is also involved in FT-induced anti-cancer immunity. The predominance of macrophages for the uptake of ablated tumors is more likely to induce a humoral response involving helper T cells and B cells rather than a cellular response (110).

### 3.4 Recruitment of immune effectors

After FT, a variety of cell types are recruited to the ablation site owing to injury response, inflammation reaction, and wound healing. These recruited cells can be immune-promoting or immune-suppressive, depending on their functional phenotype, as summarized in **Table 4**. FT can either promote or suppress anti-tumor immune responses, depending on the population and phenotype of recruited immune cells. Here, we consider immune effectors to be tumor-infiltrating immune cells that support immune recognition and cytotoxic function. The most important populations of immune effectors are tumor-infiltrating lymphocytes (TILs).

TILs including cytotoxic (CD8+) and helper (CD4+) T cells, B cells, and NK cells are emerging as prominent biomarkers in predicting the efficacy and outcome of treatments (138). Tumor-infiltrating CD8+ T cells have been shown to be positively associated with improved cancer prognoses after various forms of FT (18). Most FTs also have been reported to increase NK cell number and/or cytotoxic function (80, 139, 140). An increase of infiltrating Th1 CD4+ T cells and CD4+ CTL has been shown to be associated with enhanced immune response following focal ablation (105, 137). Tumor infiltrating B cells have been shown to positively correlate with overall survival in several solid tumors given its antigen presentation and tumor-killing function (141–143). Although the study of B lymphocytes in the context of FT is not common, there is evidence that HIFU ablation induced distinctive infiltration of B lymphocytes and RFA can lead to a change in the level of Ig secreted by B cells (144, 145).

In addition to TILs, DCs and M1 macrophages are also considered to be critical immune effectors contributing to the activation of TILs. Much evidence suggests that FT can promote DC activation and maturation and macrophage polarization toward M1 (10, 129, 146–148). Note that the historical and simplistic M1/M2 classification has the limitation of describing this transcriptionally dynamic cell type, as mixed phenotypes or populations with different phenotypes coexist. Neutrophil recruitment in FT-treated tumor due to injury response is commonly observed (149). However, little is known about the shift of neutrophil phenotypes (N1 or N2) (150) following FT.

### 3.5 Modulation of immunosuppressive cell types

Many cell types contribute to the generation of an immunosuppressive tumor microenvironment, including cancer-associated fibroblasts, myeloid-derived suppressor cells (MDSCs), Tregs, and tumor-associated macrophages (TAMs) (151). These cells are generally immune suppressive and protumorigenic. Theoretically, the elimination or reduction of immunosuppressive cell types present in the tumor bed and/or the disruption of tissue barriers can promote tumor infiltration by cytotoxic T lymphocytes, enhance anti-tumor immunity, and foster the formation of immunological memory (16).

Conflicting results have been published on how FT modulates these immunosuppressive cell types, especially for thermal ablation techniques such as RFA. For example, a number of studies have reported the reduced frequency of Treg cells in the tumor and peripheral blood after RFA, thereby promoting antitumor immunity (118, 139, 152, 153). On the contrary, some studies have shown RFA leads to the generation of an immune-suppressive environment, where immunosuppressive cell types are stimulated to proliferate, leading to tumor progression and/or aggressive recurrence (154, 155). One study pointed out that RFA leads to upregulated IL-10 and TGF-β levels, followed by a profoundly increased frequency of Treg cells in peripheral blood in a murine HCC model (156). The difference may rely on the details of RFA and the nature of the tumor.

Other modes of FTs have been more consistent in showing a decrease of immunosuppressive cell types. For instance, IRE decreases Treg cells and MDSC in the tumor bed as well as in peripheral blood (73, 83, 84). Cryoablation has also been demonstrated to induce a decrease of intratumoral Treg cells and MDSCs both *in vivo* and clinically (157). Cryo-thermal therapy can cause an elevated extracellular release of Hsp70 to induce differentiation of MDSCs into mature DCs, contributing to the relief of MDSC-mediated immunosuppression and ultimately the activation of a strong anti-tumor immune response (158).

### 3.6 Maintenance of memory cells

The maintenance of memory cells involved in local immunosurveillance is important to initiate a long-lasting antitumor immune response (16). Tissue-resident memory T cells (T_RM_), a subset of memory CD8+ T cells, are non-recirculating tissue-localized cells crucial for protective immunity against tumor recurrence (159). CD8+ T_RM_ and CD8+ T cells with a T_RM_ phenotype are typically associated with an improved prognosis in the immune response to cancer (159–164). For instance, skin T_RM_ in local immunosurveillance has been shown to be able to promote long-term protection (165). In addition, memory and memory-Like NK cells also possess traits of immunological memory against pathogens and malignancy (166).

However, little is known about how FT contributes to the establishment of endogenous tumor-specific memory cells. A study using TRAMP-C2 prostate cancer model demonstrated an increase of non-recirculating tumor-specific CD8+ T cells in non-lymphoid tissue (NLT) distal to the tumor after IRE treatment, specifically within the salivary gland, contralateral skin, and liver (167). It remains unclear, though, whether NK cells can be modulated by focal ablation. Further studies investigating the function of memory T cells are warranted.

### 3.7 Additional immunological aspects following Focal therapy

In addition to these above-mentioned 6 aspects of immune response to FT, FT can also modulate the immune system through other pathways, including but not limited to (1) cell infiltration and permeability, such as changes in vascular structure and blood flow and stroma remodeling; (2) modulation of gene expression of cancer cells and immune cells; (3) changes in metabolism, such as tumor hypoxia; and (4) tissue damage and remodeling.

It has been shown that changes in temperature can cause a wide range of changes to the tumor microenvironment. For example, hyperthermia is usually accompanied by increased blood flow, resulting in increased oxygenation and intense infiltration of inflammatory cells and tumor-infiltrating lymphocytes (TILs) (10, 16). Hyperthermia also increases the surface expression of MICA and MHC-I on tumor cells, making tumor cells more sensitive to lysis by NK cells and CD8+ T cells (168, 169). Hyperthermia also stimulates the functional activity of NK cells, macrophages, and dendritic cells (168). Non-thermal ablation techniques such as IRE have also been shown to modulate the immunosuppressive tumor microenvironment to relieve intratumoral hypoxia, which suppresses immune cells. Several components of the fibrotic stroma were also downregulated, facilitating the infiltration of cytotoxic T lymphocytes (170).

## 4 Focal therapy combined with immunotherapy

Combining FT with immunotherapy may improve the potential of focal therapy to eliminate established tumors and prevent tumor recurrence. By taking advantage of focal ablation’s ability to induce the activation of an anti-cancer immune response, the efficacy of immunotherapy can be improved. Here, we examine different types of cancer immunotherapies and the status of preclinical and clinical research combining the two forms of cancer treatment.

### 4.1 Cancer immunotherapy

Cancer immunotherapies basically work through stimulating effector mechanisms and/or counteracting inhibitory and suppressive mechanisms. The NIH categorizes immunotherapy into 5 types: monoclonal antibodies, treatment vaccines, immune system modulators, T cell transfer therapy, and immune checkpoint inhibitors (171), as summarized in **Table 5**. Immunotherapy has emerged as the fourth pillar of cancer treatment, alongside surgery, radiation, and chemotherapy.

**Table 5:** Categories of cancer immunotherapy and their functions.

Category	Function
Monoclonal antibody	• Binds to cancer cells so that the immune system will better recognize and destroy target cells • Targets signaling pathways involved in tumorigenesis. • Antibodies can function as an antagonist, which blocks the signaling pathway, or agonist, which activates the pathway.
Treatment vaccine	• Introduces tumor antigens to be taken up by APCs that in turn prime and boost the anti-tumor immune response • Oncolytic virus: infects and breaks down cancer cells without harming normal cells.
Immune system modulator	• Enhances the body’s immune response against cancer • Stimulates the immune system • Includes cytokines and immunomodulatory drugs (biological response modifiers)
T-cell transfer	• Includes TIL therapy and CAR T-cell therapy • TIL therapy: selects and collects T cells from patients, expands and infuses them back to patients • CAR T-cell therapy: chimeric antigen receptors (CARs) allow the T cells to attach to specific proteins on the surface of the cancer cells, improving their ability to attack the cancer cells
Immune checkpoint inhibitor	• A type of monoclonal antibodies (mAbs) that stimulate T cell function by blocking immune checkpoints to allow for the T cell repertoire to proliferate, become activated, and exert cytotoxic function

### 4.2 Current state of focal therapy combined with immunotherapy

The immune responses after focal ablation monotherapy are usually weak and rarely induce clinically relevant antitumor effects. Different modalities of immunotherapy have been combined with focal therapy to stimulate a more robust anti-tumor reaction with the hope of a systemic immune response, as summarized in **Table 6**. The majority of cancer immunotherapies combined with FT function through stimulating immune response components to augment anti-tumor immunity. Many proof-of-principle, animal, or preclinical studies have shown promising results, demonstrated by the improvement of DC number and/or function, increase of tumor-specific CTL response, tumor growth suppression, enhanced survival, and even inhibited metastasis (105, 157). Hundreds of clinical trials are in progress to test this combinatorial therapy in various cancer types for patients. So far, most publications have been limited to safety and tolerability. For the translation of the results seen in animals and small groups of patients, larger prospective trials need to be designed to first examine safety and efficacy before moving into randomized controlled settings. Finally, large-scale randomized controlled trials are needed to determine the clinical benefit of combined treatment.

**Table 6:** Selected examples of studies combining immunotherapy (except for ICIs, which is discussed in section 6) and focal therapy.

Immunotherapy		Focal Therapy	
Category	Therapeutic agents	Preclinical	Clinical
Treatment vaccines	DC	Cryo (172), RFA (173), IRE (174), HIFU (175)	Cryo (176), RFA (177)
Adoptive cell transfer	CAR-T cells	PTT (178)	
	CIK cells		RFA (179), Cryo (150)
	NK cells	Cryo (180)	RFA (179), IRE (181), Cryo (150)
	γδ T cells		RFA (179), IRE (182), Cryo (150)
	Th1 memory cells		Cryo (150)
Immune system modulators	GM-CSF	Cryo (183), MWA (184), RFA (184), PTT (185), IRE (186)	Cryo (187), RFA (187)
	TNF-α	Cryo (188)	
	IL-2	RFA (189)	
	IL-7 and IL-15	RFA (190)	
	CpG ODN (TLR9 agonist)	Cryo (191), PTT (192), HIFU (146)	
	Imiquimod (TLR7 agonist)	Cryo (72), PTT (193)	Cryo (194), PTT (195)
	Saponin	Cryo (196)	
	Polysaccharides, LPS	Cryo (197), PTT (198)	
	Glycated chitosan	RFA (199), HIFU (200), PTT (201)	PTT (202)
	Dinitrophenyl (hapten)		PTT (203)
	1-MT (IDO inhibitor)	PTT (204)	
	BCG	RFA (205)	
	OK-432	RFA (206), MWA (207)	
	STING agonist	IRE (208)	
Monoclonal antibody	Bevacizumab (anti-VEGF)	RFA (209)	Cryo (210)
	Anti-CD25	Cryo (113), RFA (113)	
	MEDI6469 (anti-OX40)		RFA (211)
	[131I] metuximab (anti-CD147)		RFA (212)
	Anti-CD44	PTT (213)	

Cryo, cryoablation; RFA, radiofrequency ablation; MWA, microwave ablation; HIFU, high intensity focused ultrasound; LITT, laser-induced interstitial thermotherapy; IRE, irreversible electroporation; PTT, photothermal therapy.

## 5 Immune checkpoint inhibitors (ICIs)

Immune checkpoints are crucial to the immune system for maintaining self-tolerance and modulating the duration and amplitude of physiological immune responses in order to prevent auto-immune disease (214). When checkpoint and partner proteins bind together, they send inhibitory signals to T cells. It is now clear that cancer cells harness immune-checkpoint pathways as an important mechanism of immune resistance (214). Therefore, checkpoint inhibitors that target these receptors or ligands hold promise as cancer treatments. Immune checkpoint blockade removes inhibitory signals to anti-tumor T cells, which enables tumor-specific T cells to overcome regulatory mechanisms and results in a broad enhancement of T cell-mediated immune responses (215).

Among identified immune checkpoints, cytotoxic T-lymphocyte-associated protein (CTLA-4) and Programmed cell death protein 1/Programmed death-ligand 1 (PD-1/PD-L1) are the most common targets in checkpoint blockade therapy. In general, it is perceived that CTLA-4 predominantly regulates early-stage T cell activation, whereas PD1/PD-L1 primarily regulates effector T cell activity within tissue and tumors (215, 216).

### 5.1 CTLA-4 pathway

During antigen recognition, T cells are activated when T cell receptors (TCRs) bind to antigen displayed in the MHC on APCs in concert with CD28:B7-mediated costimulation (216). CTLA-4 is a CD28 homolog with much higher binding affinity for B7 ligands (217). In resting T cells, CTLA-4 is an intracellular protein; however, after TCR ligation and a costimulatory signal through CD28, CTLA-4 expression is upregulated by translocation to the cell surface and decreased internalization (216). CTLA-4 outcompetes CD28 for binding to critical costimulatory molecules (B7-1/B7-2, also called CD80 and CD86) on APCs and mediates inhibitory signaling into the T cell, resulting in arrest of both activation and proliferation (215). CTLA-4 is also expressed by Tregs constitutively and contributes to their inhibitory functions (217). Anti-CTLA-4 antibodies block the activation of the CTLA-4 pathway, allowing for the activation and proliferation of more T-cell clones and reducing Treg-mediated immunosuppression (215, 217, 218).

### 5.2 PD-1/PD-L1 pathway

PD-1 is a member of the B7/CD28 family of costimulatory receptors (217). It regulates T-cell function through binding to its ligands, programmed death ligand 1 (PD-L1), and programmed death ligand 2 (PD-L2), which are widely expressed in nonlymphoid tissues such as cancer cells, macrophages, and myeloid cells (215). PD-1 expression on T cells is induced when T cells become activated. Upon engagement with PD-L1 and PD-L2, PD-1 is thought to primarily transmit a negative costimulatory signal through the tyrosine phosphatase SHP2 to attenuate T-cell activation and hinder cytolytic capacity (219). PD-1 expression is a hallmark of “exhausted” T cells (217), which are effector T cells with overexpressed inhibitory receptors and decreased cytokine production and cytolytic activity (220). Unlike CTLA-4, which is confined to T cells, PD-1 is also expressed in non-T lymphocyte subsets, including B cells and NK cells (214). PD-1 pathway blockade restores the activity of anti-tumor T cells that have been turned off, thus boosting an effective immune response (215, 217). Additionally, it has been pointed out that PD-1 is highly expressed on Treg cells, where PD-1 blockade actually amplifies Treg cells thus opposing the immune benefits (221).

### 5.3 FDA approved ICIs

With ample evidence suggesting that T cell response modulated by checkpoint blockade can promote durable cancer remission, the US Food and Drug Administration (FDA) has approved nine ICIs for targeted diseases (222), as summarized in **Table 7**.

**Table 7:** FDA approved immune checkpoint blockade.

Drug Name	Brand name	Target	First FDA Approval date	Cancers
Ipilimumab	Yervoy	CTLA-4	Mar-11	Melanoma, RCC, MSI-H or dMMR CRC, HCC, NSCLC, pleural mesothelioma
Pembrolizumab	Keytruda	PD-1	Sep-14	Melanoma, NSCLC, SCLC, HNSCC, Hodgkin Lymphoma, urothelial, MSI-H or dMMR cancer, MSI-H or dMMR CRC, TMB-H cancer, gastric, esophageal, cSCC, cervical, PMBCL, MCC, RCC, HCC, endometrial carcinoma, TNBC
Nivolumab	Opdivo	PD-1	Dec-14	Melanoma, NSCLC, pleural mesothelioma, RCC, Hodgkin lymphoma, HNSCC, urothelial, MSI-H or dMMR CRC, HCC, esophageal, gastric, gastroesophageal junction
Atezolizumab	Tecentriq	PD-L1	May-16	Urothelial, NSCLC, SCLC, HCC, melanoma, ASPS
Avelumab	Bavencio	PD-L1	Mar-17	MCC, urothelial, RCC
Durvalumab	Imfinzi	PD-L1	May-17	NSCLC, SCLC, BTC
Cemiplimab	Libtayo	PD-1	Sep-18	cSCC, BCC, NSCLC
Dostarlimab	Jemperli	PD-1	Apr-21	dMMR endometrial, dMMR solid
Relatlimab	Opdualag*	LAG-3	Mar-22	Melanoma

MSI-H, microsatellite instability high; dMMR, mismatch repair-deficient; TNBC, triple-negative breast cancer; cSCC, cutaneous squamous cell carcinoma; TMB-H, tumor mutation burden high; CRC, colorectal cancer; SCLC, small cell lung cancer; RCC, renal cell carcinoma; MCC, Merkel cell carcinoma; HCC, hepatocellular carcinoma; PMBCL, primary mediastinal large B cell lymphoma; HNSCC, head and neck squamous cell carcinoma; NSCLC, non-small cell lung cancer; ASPS, alveolar soft part sarcoma; BTC, biliary tract cancer. Information on approvals was gathered from Drugs@FDA.

*Opdualag is the fixed-dose combination of nivolumab and relatlimab-rmbw.

### 5.4 Emerging inhibitory checkpoints

In addition to the well-known CTLA-4 and PD-1 pathways, other immune checkpoints such as LAG-3, TIM-3, TIGIT, and VISTA are considered as promising immune therapy targets, and numerous antibodies have been developed to regulate these pathways (223, 224). Recently, the USFDA approved the combination of Nivolumab and the first lymphocyte-activation gene 3 (LAG-3)-blocking antibody, Relatlimab (BMS986016), as treatment for patients with unresectable or metastatic melanoma (**Table 7**) (225). Opdualag is also being studied in clinical trials of other cancers, including lung, colorectal, and liver cancer (226). Encouraged by the success of Relatlimab, additional drugs targeting LAG-3 and other novel immune checkpoints are being evaluated for the treatment of multiple types of cancers. We present a list of drugs that have entered clinical trial (223, 227–230) in **Table 8**. As of the writing of this review, hundreds of clinical trials are being carried out to evaluate the efficacy of these novel ICIs as monotherapy or in combination with currently approved ICI drugs.

**Table 8:** Current ICI drugs in clinical trials (not yet approved).

Checkpoints	Cells expressing target	Antibodies
LAG-3	Activated T cells, NK cells, B cells, plasmacytoid DCs	IMP321, LAG525, MGD013
TIM-3	IFN-γ producing CD4+ Th1, CD8+ T cells, Tregs, innate immune cells	TSR-022, LY3321367, MBG453
TIGIT	T cells, NK cells	OMP-313M32, MTIG7192A/RG6058
VISTA	TILs	JNJ-610588, CA-170
CEACAM1	B cells, T cells, NK cells, myeloid cells	CM-24 (MK-6018)
B7-H3	Activated T cells, NK cells, APCs	MGA271, MGD009, I-8H9
CD96	T cells, NK cells	GSK6097608
BTLA	T cells, B cells, NK cells	TAB004/JS004
KIRs	NK cells, a minority of T cells	Lirilumab, IPH2101, IPH4102
CD94/NKG2A	NK cells, a subset of CD8+ T cells	IPH2201 (monalizumab)

LAG-3, lymphocyte-activation gene 3; TIM-3, t-cell immunoglobulin mucin-3; TIGIT, t-cell immunoreceptor with immunoglobulin and ITIM domains; VISTA, V-domain Ig suppressor of T cell activation; CEACAM1, carcinoembryonic antigen-related cell adhesion molecule 1; BTLA, B- and T-lymphocyte attenuator; KIRs, killer-cell immunoglobulin-like receptors.

ICIs can be used in combination for better efficacy and response by targeting different checkpoints simultaneously. The combination of CTLA-4 and PD-1/PD-L1 blockers has been the most extensively researched regimen (231). For example, the combination of ipilimumab plus nivolumab has been approved by the USFDA for the treatment of advanced melanoma, advanced RCC, MSI-H/dMMR metastatic colorectal cancer, HCC, metastatic NSCLC, and malignant pleural mesothelioma (232). In addition to therapies utilizing CTLA-4 and PD-1/PD-L1 blockade, other combinations targeting multiple checkpoints such as LAG-3/PD-L1, TIM-3/PD-1, B7-H3/CTLA-4, and VISTA/PD-1 are undergoing pre-clinical studies and clinical trials (233).

Our knowledge of the fundamental biological roles of these molecules remains limited and, in many cases, is being outpaced by clinical investigation. Deeper understanding of the basic biology is in urgent need for the rational development of new immune checkpoint blockade therapies and combinatory approaches. Meanwhile, ongoing efforts in preclinical and clinical studies are expected to reveal mechanisms of using these novel ICIs to enhance anticancer immunity.

### 5.5 Limitations of ICI monotherapy

Although ICIs have been used widely and show great promise for improving the outcome of cancer treatment, this therapy is limited by low response rate. Many patients exhibit innate resistance and disease progression. For example, among all cancer types, anti-CTLA-4 drugs have the highest objective radiographic response of 15% in advanced metastatic melanoma (216). Anti-PD-1 ICIs (Pembrolizumab and nivolumab) have high response rate (about 80%) in Hodgkin’s disease, but intermediate response (10-45%) in melanoma, non-small cell lung carcinoma (NSCLC), bladder and urinary tract cancer, and triple-negative breast cancer (TNBC) patients (216, 219).

Immune-related adverse events (irAEs) associated with ICIs is another concern for clinical use, due to overactivation of the immune system in almost any organ of the body. IrAEs can occur at any point along a patient’s treatment course. The most common toxicities during the treatment using CTLA-4-blocking antibodies include enterocolitis, inflammatory hepatitis, and dermatitis. The most common adverse events of anti-PD-1 ICIs are fatigue, diarrhea, rash, and pruritus (216, 234, 235). Serious organ inflammatory toxicities that can be life-threatening are uncommon, but this risk may be higher with combination ICI treatments. ICI toxicity can be mitigated by high doses of corticosteroids to suppress the immune response, but these in turn can attenuate the anti-cancer immunity (216, 234).

Therefore, the evaluation of biomarkers that can predict tumor response to ICI treatment is necessary to avoid overtreatment of ICIs and minimize side effects. Emerging research has focused on identifying predictive markers and combinatory approaches to improve the relatively low response rate of ICIs (236–238).

### 5.6 Biomarkers to predict ICI therapy response

There are a wide range of biomarkers being used to predict the ICI therapy response. Tumor mutational burden (TMB, the number of somatic, coding, base substitution, and indel mutations per megabase of genome examined) is one of the biomarkers (237). For colorectal cancer (CRC), anti-PD-1 and anti-PD-L1 therapy often generates a durable response in patients with mismatch repair-deficient/microsatellite instability high (dMMR/MSI-H) CRC, which is a tumor subtype with high TMB, compared to failure in microsatellite stable (MSS) CRC, which has a significantly lower TMB (239, 240). Yarchoan et al. have analyzed 27 tumor types or subtypes and observed a significant correlation between the tumor mutational burden and the objective response rate to anti-PD-1 or anti-PD-L1 therapy (P<0.001) (237).

Certain cell surface markers have also been identified as biomarkers for ICI responsiveness. For example, PD-L1 expression has been vital to predicting tumor response to anti-PD-1 or anti-PD-L1 antibodies in melanoma and NSCLC (241, 242). PD-L1 expression is currently used to guide treatment decisions and regulatory approval. However, its expression can be upregulated by several factors including interferon gamma (IFN-γ) (243) and may vary over time and across multiple tumor sites. Upregulation of other inhibitory molecules such as TIM-3, LAG-3, and VISTA can also cause resistance to anti-PD-1/L1 antibody therapy (244). A study examining 45 FDA ICI approvals between 2011 and 2019 across 15 tumor types found that PD-L1 expression was predictive of response to PD-1/L1 ICI in only 28.9% of cases (245), suggesting the complexity and difficulty of identifying putative protein and epigenetic markers for patient stratification and potential therapeutic targeting.

Immune cell infiltration is another important predictive biomarker considering the mechanism of ICI therapy. Generally, a higher number of tumor-infiltrating lymphocytes (TILs) has been a favorable prognostic factor in many types of cancers, including melanoma and colorectal cancer (246, 247).

Taken together, “hot tumors,” characterized by high tumor mutational burden, increased expression of PD-L1 and IFN-γ signaling, and high T-cell infiltration are associated with better ICI efficacy (248, 249). In contrast, for patients with “cold” tumors such as prostate, pancreas, and brain cancers, checkpoint inhibitors are typically ineffective (249).

### 5.7 The basis of combinatory approach with focal therapy

To improve the benefit of ICI immunotherapy, especially to increase the objective response rate and reduce irAEs, substantial efforts are focused on combination strategies aimed at turning “cold” tumors into “hot” tumors (249) by changing the immunosuppressive TME or targeting other pathways that potentially inhibit the activation of T cells (250).

**Figure 2:**
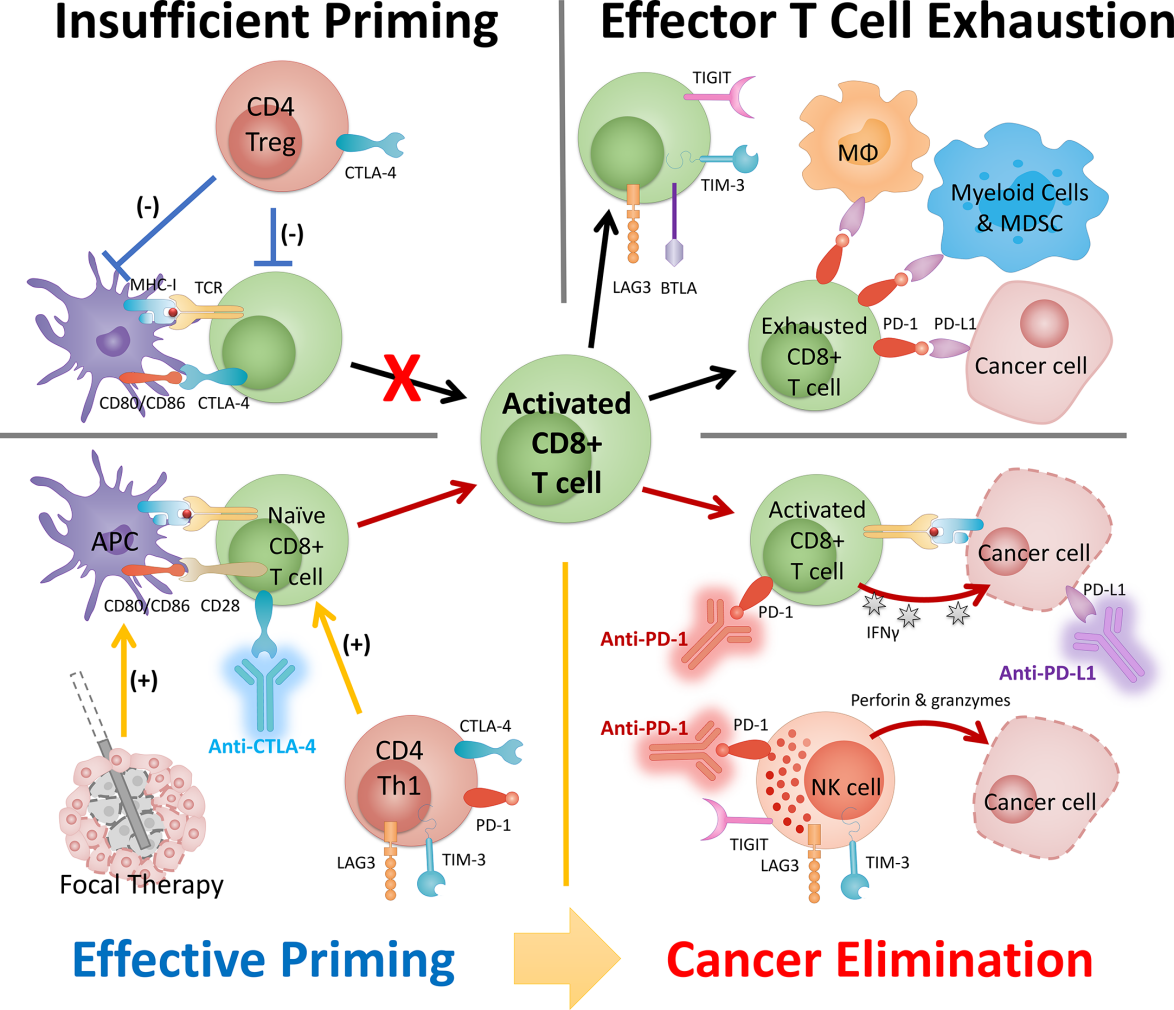
The synergy between FT and ICI serves as the basis of a combinatory approach in cancer management. The success of this combinatory strategy relies heavily on the activated CD8 T cells (i.e., population, location, and phenotype). The status of CD8 T cells is dictated by the cancer property (e.g., tumor type) and disease status and can be strongly modulated by intervention. Insufficient priming can be addressed by focal therapy and early intervention of anti-CTLA-4 and other ICI targeting Th1 CD4 T cells. T cell exhaustion can be mitigated by careful engineering of FT and ICIs (primarily anti-PD-1/PD-L1) that targets pathways preventing T cells to eliminate cancer. MDSC, myeloid-derived suppressor cells; MΦ, macrophage.

Immunomodulation by energy-based focal therapy, as we discussed previously, is well aligned with the strategy of combination therapy with ICI to improve tumor immunogenicity (**Figure 2**). In the following sections, we will discuss preclinical study and clinical trials that utilize this principle and the process in fulfilling the synergy between FT and ICI.

## 6 Preclinical *in vivo* research combining focal therapy and ICI

### 6.1 Overview of preclinical approaches

The keywords, database, and inclusion criteria for the literature research are summarized in **Supplemental Table S1**. A wide range of focal therapies have been combined with ICI therapy in various cancer models in preclinical studies. Melanoma (B16 and its derivatives), breast cancer (4T1 and NDL), HCC (Hepa1-6, H22, etc.), colorectal cancer (CT26 and MC38), prostate cancer (TRAMP, RM-1, and MycCap), and pancreatic cancer (KPC and its variants, KRAS*, etc.) are the most used cancer models to evaluate the benefits of combination therapy. Almost all the cancer models are syngeneic to corresponding mice strains (e.g., C57BL/6, BALB/c, and A/J) and were introduced by injecting cancer cells to form orthotopic or subcutaneous tumors.

The most targeted checkpoints are CTLA-4 and PD-1. ICI monoclonal antibodies, almost all purchased exclusively from Bio X Cell, are typically administered through intraperitoneal injection after FT at 100 or 200 µg per mouse for 3 or 4 total injections (**Supplemental Table S2**).

To evaluate the performance of combination therapy, researchers usually monitor the growth of the primary tumor and re-challenge long-term surviving mice with the same and/or different tumor cells to see whether this approach could improve primary tumor control and induce long-term protection against specific cancers. Some groups have also used a metastasis model or inoculated contralateral tumor to show that the combinatorial approach could enhance abscopal effect. Following the course of treatment, assessment of the immunomodulatory effect typically focuses on the CD8+ and CD4+ T cells in the tumor, spleen, and lymph nodes. Immunosuppressive cells and cytokines are also analyzed, as summarized in **Supplemental Table S2**.

### 6.2 Summary of preclinical findings

Published preclinical results suggest that FT combined with ICI is a promising treatment approach for multiple pre-clinical cancer models, as summarized in **Table 9**. Most of the research achieved 2 out of 3 of the following: (1) improved primary tumor control, (2) protection from tumor re-challenge, and/or (3) enhanced abscopal effect. It is worth noting that in all the cancer models, complete elimination of primary and systemic cancer is not guaranteed by monotherapy, but the chances have been greatly increased by the combinatory approach.

**Table 9:** Materials and methods of combinatory FT and ICI in preclinical models. Y, results observed. ―, no data.

Cancer model	ICI	Improve primary tumor control	Rechallenge Protection	Enhance abscopal effect	Reference
Radiofrequency ablation					
B16-OVA	Anti-CTLA-4	―	Y, 75% rejection	―	(22)
B16-OVA	Anti-CTLA-4	―	Y, ~80% rejection	―	(191)
CT26	Anti-PD-1	Y	Y, 80% rejection	Y	(251)
CT26, MC38	Anti-PD-1	Y	―	―	(154)
CT26	Anti-CTLA-4	Y	―	―	(252)
H22	Anti-CTLA-4	Y	Y, 75% rejection	―	(253)
H22	Anti-CTLA-4	Y	Y, 60% rejection	―	(254)
Microwave ablation					
Hepa 1-6	Anti-CTLA-4	Y	Y, 90% rejection	Y	(184)
4T1	Anti-PD-1 + Anti-CTLA-4	Y	Y, 80% rejection	―	(255)
Hepa 1-6	Anti-PD-1 + Anti-CTLA-4	Y	Y, 67% rejection	―	(256)
Hepa 1-6	Anti-PD-1	Y	Y, 60% rejection	Y	(257)
MC38	Anti-LAG3	Y	―	―	(258)
High intensity focused ultrasound					
NDL	Anti-PD-1	Y	―	Y	(259)
NDL	Anti-PD-1	Y	―	Y	(81)
CT26	Anti-CTLA-4	Y	Y, 80% rejection	Y	(252)
4T1	Anti-PD-1	Y	―	―	(260)
NDL	Anti-PD-1	Y	―	―	(146)
KPC	Anti-CTLA-4 + Anti-PD-1	Y	―	―	(261)
Cryotherapy					
B16-OVA	Anti-CTLA-4	Y	Y	―	(113)
TRAMP-C2	Anti-CTLA-4	Y	Y, 44% rejection	―	(262)
RM-1	Anti-CTLA-4	Y, inhibit metastasis	―	―	(263)
B16-OVA,CT26	Anti-CTLA-4	Y	―	Y	(264)
MycCap	Anti-PD-1 or Anti-CTLA-4	Y	Y	―	(265)
RENCA (RCC)	Anti-PD-1	Y	Y	―	(266)
MMT-060562 (breast cancer)	Anti-PD-1	―	―	Y	(267)
H22	Anti-PD-L1	Y	―	―	(268)
4T1	Anti-PD-1	Y	―	Y	(269)
Irreversible electroporation					
KPC4580P	Anti-PD-1	Y	Y	Y	(270)
KRAS*	Anti-PD-1	Y	Y, 100% rejection	―	(170)
Hepa 1-6	Anti-PD-L1	Y	―	―	(271)
TRAMP-C2	Anti-CTLA-4 or Anti-PD-1	Y	Y, 100% rejection	―	(167)
EG7 (lymphoma)	Anti-PD-L1	Y	―	Y	(272)
Hepa 1-6	Anti-PD-L1	―	―	―	(273)
Mechanical destruction (histotripsy, M-HIFU etc.)					
CT26	Anti-PD-1	Y	Y	―	(274)
Neuro2a (neuroblastoma)	Anti-CTLA-4 + Anti-PD-L1	Y	Y, 100% rejection	Y	(275)
4T1	Anti-PD-1	Y	―	―	(276)
B16GP33, Hepa1-6	Anti-CTLA-4	Y	―	―	(277)
MM3MG-HER2	Anti-PD-L1	Y	Y	Y	(278)
TRAMP-C2	Anti-PD-1	Y	―	―	(61)
Hyperthermia					
4T1	Anti-CTLA-4	Y	Y, 100% rejection	Y	(279)

The induced immune response of thermal ablation, most commonly RFA and MWA, is considered incapable of complete eradication of established tumors or durable prevention of disease progression (130). With the addition of ICI, positive prognostics are often observed. Anti-CTLA-4 and RFA in the B16-OVA model have been shown to augment the anti-tumor effect of splenocytes, which resulted in long-lasting tumor protection (22) and regulatory T cell depletion in addition to increased tumor-specific T cell numbers, therefore protecting mice from tumor challenges (113). Anti-PD-1 in tandem with RFA in a colon cancer model has been reported to result in stronger anti-tumor immunity, demonstrated by prolonged survival and reversed immunosuppression in distant lesions (251). Combined ICI and MWA share similar trends: anti-CTLA-4 co-administered with MWA and GM-CSF contribute to 90% secondary tumor rejection in Hepa 1-6 model mice (184). Further studies revealed that the tumor-specific augmentation is supported by NK, CD4+, and CD8+ T cells (255, 256).

Some successes have also been reported on HIFU working well with ICI immunotherapy. Extended survival with partial tumor ablation, abscopal effects, and inhibition of re-challenged tumors have been reported. These observations are correlated with systematically activated DCs and TILs and downregulated Tregs (252, 275).

In contrast to thermal ablation such as RFA and MWA, which relies on a temperature typically above 60 ˚C, local hyperthermia (42.5 ˚C for 20 min) with anti-CTLA-4 was also shown to enhance the anti-tumor response (279). Other studies generate local hyperthermia using photo-absorber dye (photothermal therapy) combined with ICI treatment to stimulate tumor-specific immune responses (35, 36). These studies, based on various heating methods, demonstrate that thermal therapy is capable of magnifying the impact of ICI immunotherapy, especially for treating “cold” tumors.

Cryoablation has been described to exert both immunostimulatory and immunosuppressive effects due to the specific physiological mechanisms of cold injury (105, 130). Some studies show that cryoablation has no effect on the growth of distant secondary tumors or on increased tumor-specific CD8 T cells (130, 262). However, the therapeutic effect of cryoablation is substantially improved with anti-CTLA-4: secondary tumors are significantly slowed or prevented (262, 263). Compared to cryoablation monotherapy, secondary tumors in the combination group were highly infiltrated by CD4 T cells and CD8 T cells, and there was a significant increase in the ratio of intratumoral T effector cells to FoxP3(+) Tregs (262).

Despite the unique advantage of IRE over thermal-based ablation, especially in pancreatic cancer, IRE alone is insufficient to eradicate remote micrometastatic lesions completely, and most patients receiving this therapy develop distant progression (280). Synergizing IRE with ICI immunotherapy to address local and systemic cancer has a huge potential for clinical translation in PDAC. In one example, IRE reverses resistance to immune checkpoint blockade in a murine orthotopic PDAC model with a long-term memory immune response (170). In another example in prostate cancer, IRE and anti-CTLA-4 increased intratumoral tumor-specific T cells and increased tissue-resident CD8+ memory T cells (T_RM_) in non-lymphoid tissues including skin. Mice that had previously achieved complete remission following dual IRE + anti-CTLA-4 therapy were also 100% protected from secondary tumor challenge (167).

The immunologic effects of mechanical ablation (including mechanical HIFU, boiling histotripsy and cavitation-cloud histotripsy) are less well characterized. This non-thermal ablation process can increase the expression of both CTLA-4 and PD-1 pathway receptors (281), which serves the basis of synergistic effect with ICI.

### 6.3 Nanoparticle-mediated hyperthermia

In recent years, the number of preclinical studies combining NP-mediated hyperthermia therapy with ICI has increased rapidly, especially with a large number of studies from China, as summarized in **Supplemental Table S3**, NP-mediated hyperthermia therapy + ICI *in vivo* research on animals. In preclinical studies, NP-mediated hyperthermia, both photothermal therapy (PTT) and magnetic hyperthermia (MHT), combined with ICI has been demonstrated to enhance therapeutic outcomes, reverse tumor-mediated immunosuppression, result in therapeutic effect for both primary tumors and metastatic lesions, prevent cancer recurrence, and prolong the survival period (282, 283).

As shown in **Supplemental Table S3**, among numerous nanoparticles used for hyperthermia therapy with ICIs, near-infrared lasers using gold nanoparticles are most common. A wide range of tumor models are used: most of them are orthotopic or subcutaneous syngeneic tumors. Among all the cancer models, the most studied is 4T1 (breast cancer), followed by CT26 (colorectal cancer) and B16 (melanoma). These 3 models make up more than 80% of the preclinical studies on the list. Different from ablative thermal therapy, where temperatures greater than 55°C are common, these NP-mediated hyperthermia therapies typically operate at a lower temperature. Most of the literature has reported enhanced tumor antigen-specific T cell responses, inhibition of Treg cell functions, promoted M1 macrophage differentiation, and improved tumor-infiltrating lymphocytes (284).

Compared to conventional hyperthermia, NP-mediated hyperthermia offers several advantages: in theory, the cancer cells can be selectively targeted and ablated at microprecision scale due to the propensity of NPs to extravasate from the tumor vascular network in some cancers and accumulate in and around cancer cells (285). The construction of NPs can be easily modified according to therapeutic needs; for example, anti-cancer drugs can be linked to or carried by NPs to enhance tumor killing and immune activation. The versatility of multifunctional NPs has enabled the integration of hyperthermia with other treatment approaches such as photodynamic therapy (PDT), chemodynamic therapy (CDT), and even immunotherapy within the same platform.

As of the writing of this review, there has been no clinical trial combining NP-mediated hyperthermia with ICI. The intrinsic limitations of both PTT and MHT are barriers preventing large-scale clinical translation from small animal models, even when combined with immunotherapy. First, uniform and fast energy deposition to the entire tumor remains challenging: the variation of NP concentration within the tissue, the limited tissue penetration depth (a few millimeters) of light for PTT and the poor magnetothermal conversion efficiency for MHT all severely dampen the treatment effect. Second, effective delivery of nanomedicine remains a major challenge. Only a fraction of intravenously administered nanomaterials can be delivered to the tumor regions, while the majority are absorbed by the reticuloendothelial system during blood circulation, followed by clearance from the body (285). At the same time, the biosafety of nanomedicines needs to be assessed systematically in order to prevent severe treatment-related side effects. Most animal studies have utilized intratumoral delivery of the NP agent to guarantee enough NPs located in the tumor, which is not always feasible for clinical cases. Third, the immunomodulatory effects of NP-mediated hyperthermia need to be mechanistically studied and understood. The success of these combination approaches appears to be linked to a combination of factors related to the hyperthermia response and specifics of each nanoparticle platform, necessitating an understanding of the mechanisms of interplay between hyperthermia and nanoparticle design.

## 7 Clinical research combining focal therapy and ICI

### 7.1 Clinical approaches of focal therapy and ICI

#### 7.1.1 Overview of clinical trials

The keywords, database, and inclusion and exclusion criteria of the literature research of clinical trials are summarized in **Supplemental Table S4**. When more than one report of the same study was available, the most recent data (with longer follow-up and/or higher number of patients) were included. 

**Figure 3:**
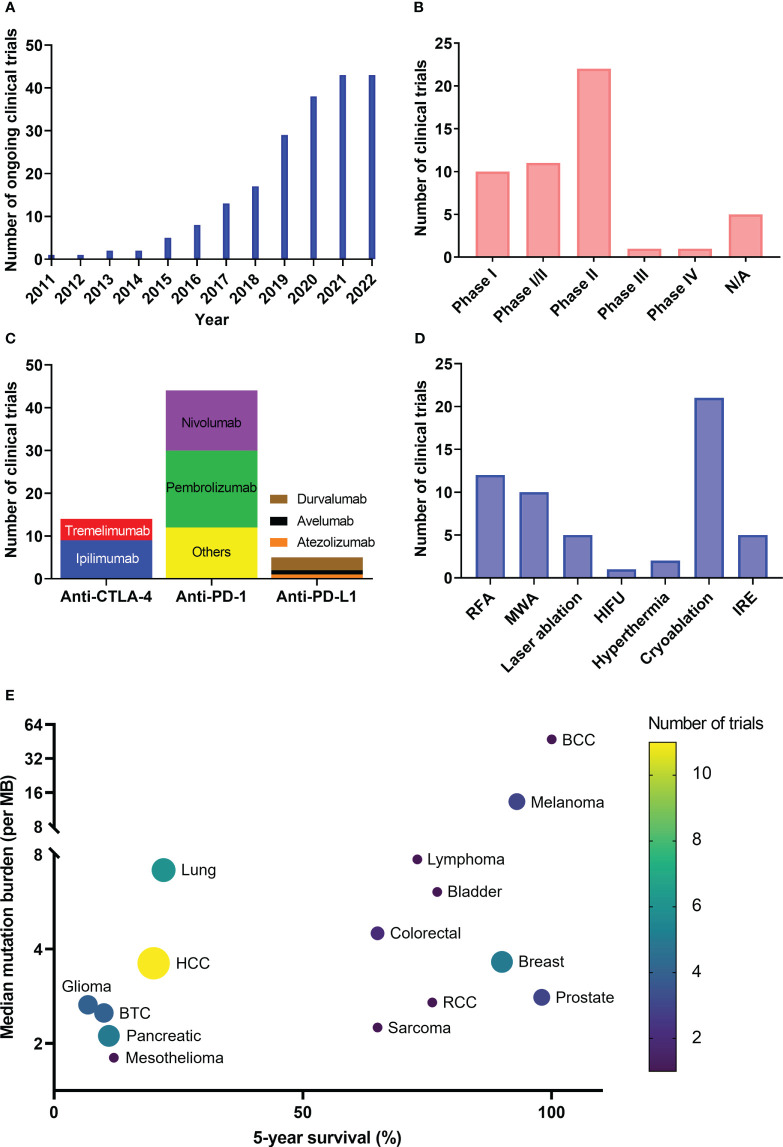
Clinical trial summary. The numbers of clinical trials are grouped by year **(A)**, clinical phase **(B)**, use of ICI drug **(C)**, focal therapy modality **(D)** and targeted diseases with their tumor mutation burden and 5-year survival **(E)**. BCC, basal cell carcinoma; BTC, biliary tract carcinoma; HCC, hepatocellular carcinoma; RCC, renal cell carcinoma.

The keyword search identified a total of 55 studies by Sept 15, 2022, as summarized in **Table 10**. Five were excluded as they were withdrawn or terminated before completion, which would not yield meaningful results. The 50 clinical trials included were sorted by FT type and ICI type, separately. In general, the number of ongoing clinical trials has increased steadily each year since 2011 (**Figure 3A**). Seven different types of FTs and 11 distinct ICIs have been used in the identified trials. Several studies involved the comparison of different FTs or ICIs, and 10 out of the 50 used more than 1 ICI in the treatment arm.

**Table 10:** Clinical trials of focal therapy combined with ICI immunotherapy.

Identifier	Ablation modality	Immunomodulator	Cancer type & stage	Study phase
NCT05302921	Cryoablation	Ipilimumab + Nivolumab	Relapsed/Refractory Pediatric Solid Tumors	II, Recruiting
NCT05277675	RFA	Tislelizumab/Sintilimab	Recurrent HCC	N/A, Recruiting
NCT05053802	MWA	Camrelizumab	Multiple Primary Lung Cancer	II, Recruiting
NCT05010668	Cryoablation	Sintilimab	Intrahepatic Cholangiocarcinoma	II, Recruiting
NCT04864379	RFA	PD-1 inhibitor	Advanced Malignant Solid Tumor	I, Recruiting
NCT04835402	IRE	Pembrolizumab	Pancreas Cancer, Metastatic	II, Active, not recruiting
NCT04805736	MWA	Camrelizumab	Breast Cancer	II, Recruiting
NCT04707547	RFA	Nivolumab	Liver Cancer	IV, Completed
NCT04701918	Cryoablation	Pembrolizumab	Metastatic Urothelial Carcinoma, Bladder Cancer	II, Recruiting
NCT04652440	RFA or MWA	Tislelizumab	HCC	I/II, Recruiting
NCT04612530	IRE	Nivolumab	Metastatic PDAC	I, Recruiting
NCT04570683	LITT	Nivolumab	Basal Cell Carcinoma	I, Recruiting
NCT04339218	Cryoablation	Pembrolizumab	Metastatic Lung Adenocarcinoma	III, Recruiting
NCT04299581	Cryoablation	Camrelizumab	Intrahepatic Cholangiocarcinoma	II, Recruiting
NCT04220944	MWA	Sintilimab	Hepatic Carcinoma	I, Recruiting
NCT04187872	LITT	Pembrolizumab	Recurrent Brain Metastasis	I, Recruiting
NCT04249167	Cryoablation	Atezolizumab	Locally advanced or metastatic TNBC	I, Withdrawn
NCT04212026	IRE	Nivolumab	Metastatic Pancreatic Cancer	II, Recruiting
NCT04201990	Cryoablation	Camrelizumab	Multiprimary Lung Cancer	I/II, not yet recruiting
NCT04156087	MWA	Durvalumab + Tremelimumab	Pancreatic Cancer Non-resectable	II, Recruiting
NCT04150744	RFA	Camrelizumab	HCC	II, Recruiting
NCT04118166	Cryoablation	Ipilimumab + Nivolumab	Metastatic or Locally Advanced Soft Tissue Sarcoma	II, Active, not recruiting
NCT04116320	HIFU	Ipilimumab	Advanced Solid Tumors	I, Recruiting
NCT04102982	MWA	Camrelizumab	NSCLC Stage IV	II, Recruiting
NCT04090775	Cryoablation	Nivolumab + Ipilimumab	Metastatic Prostatic Adenocarcinoma	II, Completed
NCT03982004	Cryoablation	Pembrolizumab	Metastatic Breast Cancer	Terminated
NCT03949153	Cryoablation	Ipilimumab + Nivolumab	Stage IIIB/C Melanoma	I/II, Completed
NCT03939975	RFA or MWA	Pembrolizumab or Nivolumab	Advanced HCC	II, Completed
NCT03873818	Cryoablation	Ipilimumab + Pembrolizumab	Metastatic Melanoma	II, Recruiting
NCT03864211	RFA or MWA	Toripalimab	HCC	I/II, Active, not recruiting
NCT03769129	MWA	Pembrolizumab	NSCLC	N/A, Recruiting
NCT03757858	Hyperthermia	Pembrolizumab	Abdominal and Pelvic Malignancies or Metastases	I/II, Recruiting
NCT03753659	RFA or MWA	Pembrolizumab	HCC	II, Recruiting
NCT03630640	IRE	Nivolumab	HCC	II, Active, not recruiting
NCT03546686	Cryoablation	Ipilimumab + Nivolumab	Triple-negative Breast Cancer	II, Recruiting
NCT03393858	Hyperthermia	Pembrolizumab	Advanced Malignant Mesothelioma	I/II, Recruiting
NCT03341806	LITT	Avelumab	Recurrent GBM	I, Completed
NCT03325101	Cryoablation	Pembrolizumab	Stage III to IV Cutaneous Melanoma	I/II, Active, not recruiting
NCT03290677	Cryoablation	Pembrolizumab or Nivolumab or Atezolizumab	Lung, Stage IV	N/A, Recruiting
NCT03277638	LITT	Pembrolizumab	Recurrent GBM	I/II, Recruiting
NCT03237572	HIFU	Pembrolizumab	Metastatic breast cancer	I, Terminated
NCT03189186	Cryoablation	Pembrolizumab	Renal Cell Carcinoma, Metastatic Kidney Cancer	I, Withdrawn
NCT03101475	RFA	Tremelimumab + Durvalumab	Colorectal Cancer, Liver Metastases	II, Completed
NCT03080974	IRE	Nivolumab	Advanced Pancreatic Adenocarcinoma	II, Active, not recruiting
NCT03035331	Cryoablation	Pembrolizumab	Non-Hodgkin Lymphoma	I/II, Active, not recruiting
NCT02833233	Cryoablation	Ipilimumab + Nivolumab	Breast Cancer, early, resectable	N/A, Active, not recruiting
NCT02821754	RFA or Cryoablation	Tremelimumab + Durvalumab	HCC, BTC	II, Active, not recruiting
NCT02626130	Cryoablation	Tremelimumab	Metastatic RCC	I, Completed
NCT02489357	Cryoablation	Pembrolizumab	Stage IV Prostate Cancer	N/A, Completed
NCT02469701	Cryoablation	Nivolumab	NSCLC	II, Terminated
NCT02437071	RFA	Pembrolizumab	Metastatic Colorectal Cancer	II, Active, not recruiting
NCT02423928	Cryoablation	Pembrolizumab	Prostate Cancer	I, Completed
NCT02311582	LITT	Pembrolizumab	Recurrent Malignant Glioma	I/II, Active, not recruiting
NCT01853618	RFA or Cryoablation	Tremelimumab	HCC, BTC	I/II, Completed
NCT01502592	Cryoablation	Ipilimumab	Breast Cancer, early stage/resectable	I, Completed

The majority of trials were in phase I or II or a combination of phase I and II, enrolling small cohorts, typically fewer than 50 patients. One phase III and one phase IV trial (both outside U.S.) were identified (**Figure 3B**). As of September 2022, more than half of the trials were still recruiting, and only 11 trials have been completed. In general, most of these studies are in the early phase, and there is indispensable need for randomized phase III trials to confirm the clinical efficacy of these novel combinations.

#### 7.1.2 ICI used in clinical combination therapy

Most of the clinical trials used anti-PD-1 drugs (Pembrolizumab or Nivolumab) or anti-CTLA-4 drugs (Ipilimumab) in combination with FT, which are the first three ICI drugs approved by the FDA. The number of ICIs that are used in combination treatment has been increasing during the past 10 years. Although ipilimumab was the only ICI involved in a clinical trial in 2011, 11 distinct ICIs have been included in clinical trials by 2022 along with the thriving development of ICI drugs worldwide (**Figure 3C**). Anti-PD-1 antibodies were the most frequently used ICIs across all studies, with Pembrolizumab included in 18/50 trials, followed by Nivolumab (14/50), due to a number of factors including the broad applications (**Table 7**), the largest market share in ICI (286) and being manufactured by numerous pharmaceutical companies globally.

Dual immunotherapy using an anti-PD-1/L1 antibody and an anti-CTLA-4 antibody with FT was identified in 10/50 trials. Ipilimumab + Pembrolizumab/Nivolumab (7/50) were the most common double-agent immunotherapies along with FT. The application of dual combinations is encouraged by the evidence that combination anti-CTLA-4 plus anti-PD-1 checkpoint blockade has shown enhanced efficacy compared to monotherapy in a wide range of cancer types (287–289). In addition, multiple studies included other immunotherapies such as CIK therapy and dendritic cell injection in order to further enhance anti-tumor immune response.

#### 7.1.3 FT used in clinical combination therapy

Cryoablation, which has been used in 21 trials (19 single FT + 2 more than one FT), has been the most commonly studied FT modality (**Figure 3D**), followed by RFA, MWA, IRE, LITT, hyperthermia, and HIFU. Also, cryoablation has been used in combination with 8 ICIs reported in this study, probably owing to the solid understanding of cryoinjury and the long history of this technique being applied in numerous diseases (**Table 2**). Among identified clinical trials, the combination of Pembrolizumab/Ipilimumab and cryoablation was most frequently studied.

The choice of FT in combination for specific disease is largely based on the established clinical benefit of locoregional therapy (LRT) monotherapy, which depends on the properties of the targeted tissue and the FT mechanism, as we reviewed in section 2. For example, RFA, MWA and cryoablations were used on primary liver tumors, while cryoablation was the most frequently studied FT modality to be combined with ICIs for primary breast cancer.

#### 7.1.4 Combination therapies benefits a broader range of cancer patients

The combination of FT and ICI is likely to benefit a broader range of patients than monotherapy, reflected by the widening scope of indications for which this treatment has been tested. In **Figure 3E**, we map the number of clinical trials on both TMB and 5-year survival for each specific cancer type. The references for mutation burden and survival data are summarized in **Supplemental Table S5**.

Combination therapies greatly expand the application of ICI, offering opportunities to treat a wider variety of cancer where ICI monotherapy is ineffective. When used alone, ICI therapy has been mostly limited due to low response rates; the FDA-approved indications focus on “hot” tumors with high TMB. Cancer types with higher response rates to ICI largely associated with their TMB, including melanoma, lung cancer, and colorectal cancer, have been treated with combination therapy. In addition, certain cancers with lower ICI response rates due to their relatively low mutation loads, such as prostate cancer, pancreatic cancer, and MSS CRC, are being treated in clinical trials with combination therapy. Still other cancer types, including HCC, BTC, and glioma are benefiting from combination therapy with ICI, mainly owing to the crucial role that LRTs have played in managing these diseases.

LRT monotherapies are usually considered in patients with unresectable local disease, as either a conservative approach (high 5-year survival, such as breast cancer and prostate cancer) or a palliative or even “last-ditch measure” (low 5-year survival, such as glioma and pancreatic cancer), according to NCCN guideline. In comparison, the combination therapies are being evaluated as a first- or second-line treatment: not limited to primary tumors but also used for recurrent and/or metastatic disease.

Taken together, combination therapy shows promise for mitigating the limitations of both ICI and FT monotherapy to expand their application. This strategy not only makes existing monotherapy more effective, but more importantly, opens up possibilities for a broader range of cancers.

### 7.2 Summary of clinical findings

It is anticipated that the combination of ICIs and FTs can produce synergistic effects, leading to improved outcomes without added toxicities. The primary reported outcomes of clinical studies examined, most commonly, safety and tolerability, survival, response rate, and immune-related biomarkers. The potential benefits of combination therapy include: 1) improved therapeutic effect, 2) enhanced immune response, and 3) reduced adverse events (**Table 11**). The outcomes of clinical reports are summarized in **Supplemental Table S6**.

**Table 11:** Clinical report summary. ★ ★: good, ★: marginal, ◎: No comparison, ―: No data.

Identifier	FT	ICI	Patient size	Improved therapeutic effect	Enhanced immune response over monotherapy	Reduced adverse events	Ref
NCT03873818	CRYO	Ipilimumab or Pembrolizumab	16	◎	―	◎	(290)
NCT03290677	CRYO	Pembrolizumab or Nivolumab or Atezolizumab	18	◎	―	◎	(291)
NCT02833233	CRYO	Ipilimumab + Nivolumab	5	◎	★ ★	◎	(292)
NCT02821754	CRYO	Tremelimumab+ Durvalumab	22	★	―	◎	(293)
NCT02626130	CRYO	Tremelimumab	29	★	★ ★	★	(294)
NCT02489357	CRYO	Pembrolizumab	12	◎	◎	◎	(295)
NCT02423928	CRYO	Pembrolizumab	18	◎	◎	◎	(296)
NCT01502592	CRYO	Ipilimumab	19	★	★ ★	★	(297)
NCT03939975	RFA or MWA	Nivolumab or Pembrolizumab	50	★★	―	★	(298)
NCT03864211	RFA or MWA	Toripalimab	48	★★	―	★	(299)
NCT02821754	RFA	Tremelimumab+ Durvalumab	58	◎	―	◎	(300)
NCT02437071	RFA	Pembrolizumab	26	◎	―	◎	(301)
NCT01853618	RFA	Tremelimumab	39 (HCC)	◎	★★	◎	(302, 303)
NCT01853618	RFA	Tremelimumab	20 (BTC)	◎	★	◎	(304)
NCT03341806	LITT	Avelumab	14	―	―	―	(305)
NCT03757858	Hyperthermia	Pembrolizumab	33	★	★ ★	★ ★	(306)
NCT03080974	IRE	Nivolumab	10	◎	◎	◎	(307)

#### 7.2.1 Improved therapeutic effect

The therapeutic effect is improved by an increase of response rate and/or better survival compared to either FT or ICI monotherapy. For example, a phase II study that evaluated subtotal thermal ablation (RFA or MWA) with anti-PD-1 therapy (nivolumab or pembrolizumab) in patients with advanced HCC has shown that additional ablation increases the objective response rate with tolerated toxicity and achieves a relatively better median survival (298). In this study, the complete response rate and partial response rate were increased from 4% to 8% and from 6% to 16%, respectively, when ablation was added to anti-PD-1 monotherapy. Another phase Ib study assessing combined IRE with nivolumab on locally advanced pancreatic adenocarcinoma showed a complete ablation effect in 100% of patients, a mean time to progression (TTP) of 6.3 months, mean progression-free survival (PFS) of 6.8 months (95% CI, 3.5-10.0), and a median overall survival (OS) of 18.0 months (95% CI, 9.2-26.8) (307). Encouraged by the remarkable efficacy, a multicenter, phase II adjuvant trial (NCT03080974) is underway evaluating IRE and nivolumab in patients with locally advanced pancreatic cancer.

#### 7.2.2 Enhanced immune response

An enhanced immune response is typically demonstrated by an elevation in the biomarkers associated with stronger anti-tumor immunity in most of the trials or lessening of immune anergy. Most noticeable is the sustained elevation in intratumoral and/or peripheral CD8 T cells in combination therapy. In one study, cryoablation and tremelimumab treatment led to a significant increase in immune cell infiltration and tertiary lymphoid structures in patients with metastatic clear cell RCC (294). Another phase I/II study using tremelimumab and loco-regional therapy (RFA, cryoablation, or TACE) on HCC patients showed an activation of tumor-specific T cells and a decrease in T-cell clonality on the patients (302). A study involving cryoablation and ipilimumab on breast cancer showed that combination therapy was associated with increases in Th1-type cytokines, an increased frequency of activated T cells and proliferating T cells in PBMC, and an expansion of effector T cells within the tumor relative to regulatory T cells (297).

#### 7.2.3 Reduce adverse effects

Safety and tolerability are the primary outcome for most studies demonstrating the reduction of adverse effects. Among all the reported trials, the combination therapy was well-tolerated, and the toxicity is not increased by the association of ICIs and LRTs. Adverse events were less than or equal to Grade II for most of the studies. Moreover, the ICI dose in combination treatment can be reduced owing to the increase in therapeutic effect and improved response rate, therefore reducing the immune-related adverse effects.

#### 7.2.4 Opportunities in clinical research

More clinical evidence is needed to demonstrate whether the combination of ICIs and FTs is feasible and effective in treating solid tumors and shows advantages over monotherapy and even SOCs. As of the writing of this review, the numbers of completed clinical trials and retrospective clinical studies are still small, especially compared to those in radiation oncology or chemotherapy. Among these studies with combination FT and ICI, the patient selection may be biased; some patients with low TMB (and presumably low response rate to ICI) may have been excluded. In addition, more detailed analysis of immune response is warranted for better understanding of immune modulation by this combined treatment.

There has not been a reported randomized phase III study that compares combination therapy with other treatments including monotherapy (FT or ICI alone). The immune and therapeutic effects of the combination therapy remain to be better understood. Fundamental questions in designing optimal combinatorial treatment regimen need to be answered, as we discuss in our next sections.

## 8 Challenges for FT and ICI

### 8.1 Lack of “immunological dosing” of FT

Unlike thermal dosing, the “immunological dosing” of FT, which describes the interaction between the immune system and the FT, is not well defined nor established. Even though there have been comprehensive reviews on the immunomodulation of types of FT such as cryoablation (110), hyperthermia (130), and IRE (308), the quantitative relationship between the exact treatment conditions and the subsequent immune responses (summarized in section 3) have not been well described. For example, even though numerous studies have demonstrated that hyperthermia could provoke robust immune responses and create an immune-favorable TME that sensitizes tumors to immunotherapy (309, 310), the relationship between hypothermia parameters (the elevated temperature, time of elevated temperature, and distribution of temperature) and the extent of immune system activation still needs to be elucidated (283, 311). To be specific, the quantity of antigen being released as a function of tumor volume and treatment condition (e.g., temperature and time for thermal ablation) is yet to be uncovered. The amount of tumor antigen needed to sufficiently prime the immune system is largely unknown, despite its well-recognized importance. In addition, the composition of DAMPs generated following FT is not clear either. Furthermore, a quantitative relationship between the dynamics of DAMPs, and specific FT treatment conditions is largely unknown. Understanding the immune impacts of FT will require careful and extensive mechanism studies that also includes variability based on heterogeneity of tumor types.

The best choice of FT is usually not straightforward. Currently, clinicians are not selecting FT approaches based on the immunological effects of specific FT modalities, in part because such immunologic effects are not yet clear, but by other factors, including the tumor location, tumor size, and disease stage. To date, clinical studies offering direct comparison of the immune response among the different modes of FT have not been conducted. Preclinical comparison among different modes of FT is also still lacking. Another factor that further complicates the issue is that the device and setup of FTs vary among different laboratories: some use a clinical-size probe which is less than ideal for sub-centimeter tumors, while some groups opt to build probes specially for small animal research (312). When FT probes for small animals are used, they are usually not fully characterized: lacking an assessment of the *ex vivo* and *in vivo* physical performance of the probes with regards to the biological sequelae of tumor destruction.

Proper dosing in clinical study is key for optimal combination therapy. For example, even though RFA and anti-PD-1 generally works well in most of the cases, the inflammation induced by incomplete RFA can accelerate tumor progression and hinder anti-PD-1 immunotherapy (154). However, studying FT dosing in humans is difficult if not impossible, so careful examination of controlled FT dosing must be studied in small animal models, like rodents, despite their difference in size. Fundamentally, the “immunological dosing” of FT on a tumor should depend on the energy field and its coverage. Even though the energy field (spatiotemporal distribution of physical quantities, such as temperature for thermal therapy and cryoablation, electric field for IRE) has been extensively studied for cell death and tissue destruction, unfortunately the importance of a well-described energy field is largely overlooked in both preclinical and clinical studies: when a tumor is being treated, the direct physical quantities are usually not quantitively prescribed or not being monitored. In addition, for example, when comparing the additive or synergistic effect of combinatory therapy to energy-based focal therapy, controllable and consistent partial ablation is desirable so that additional therapeutic benefit can be quantitatively analyzed by tracking the tumor volume over time from the same baseline. However, such a baseline is not easy to achieve without controlling the energy field. To delineate the energy field of a specific FT, a few steps have to take place (1): the device needs to be well-designed for repeatable delivery of the energy (2), the energy needs to be precisely mapped based on the boundary condition defined by the device and the multi-physics models, and (3) *in vivo* measurements including monitoring and validating the energy fields need to be taken and compared to the models to ensure the consistency of FT protocols.

### 8.2 No consensus on the timing of ICI when combined with FT

Despite research advancement toward understanding synergistic combination therapy, the optimal timing of ICI treatment has not been well studied. Little is known about how to design the combinatory regimens to favor therapeutic efficacy, whether before, during or after the FT. Most clinical studies incorporated FTs during ICI treatment. This fact might lead to weakened responses, as some preclinical evidence suggests that checkpoint inhibition before antigen priming can lead to poor anti-tumor effects (313). A popular theory is that ablation should be performed before ICIs because neoantigen-specific T cells induced by interventional therapy can have an improved response to ICI, thus enhancing the anti-tumor immune response. However, some researchers propose that administering ICI therapies should be in a neoadjuvant setting prior to FT (314) and argue that immunotherapy begun before ablation can be curative and can enhance efficacy in the presence of a high tumor burden (259). Moreover, the immunomodulatory effects produced by some ablation treatments may be short-lived, so the timing of sequential treatment of ICIs needs careful planning. Unfortunately, to knowledge, no study was designed with this issue in mind. There is indispensable need for preclinical study to answer the question of how the sequencing of FT and ICI affects optimal anti-tumor response.

In addition to timing, there is a need for preclinical and clinical investigation to optimize the synergistic efficacy of the dosage, frequency, and delivery approach of ICI when combined with FT. Most of the studies adopted dosage regimens based on the previous experience of preclinical studies or previous clinical trials on ICIs alone and adjusted them according to regular evaluation (315), rather than a rigorous optimization process. ICI usually comes with dose-limiting immune-related side effects; these side effects may be mitigated by incorporating FT to enhance the response of ICI, as we summarized previously.

### 8.3 Gaps between preclinical models and clinical trials

Most research in this field is currently still in the proof-of-principle phase. Preclinical research largely precedes clinical studies in terms of optimizing treatment approaches and understanding immune system modulation. However, we should recognize the gaps between preclinical models and clinical trials, especially considering differences in (1) the cancer models (2), the size difference, and (3) the drug delivery approaches.

The immune effect is highly dependent on not only the cell death by FT, but also the type of tumor in the experimental mouse model. The extent to which preclinical research can be generalized to humans is largely limited by the heterogeneity of tumor types, animal models, and ablation methodologies. The mouse subcutaneous tumor model has prominent advantages, allowing treatment of a large number of animals with easy handling and housing and lower costs than other mammalian species, and also offering access to an array of assays, reagents and genetically engineered mice that facilitate mechanistic understanding (70). However, there is divergence between mouse and human immune systems, and the immune microenvironment of subcutaneous tissue is significantly different from those in other organs. In addition, the inoculated tumor model using syngeneic cancer cell lines cultured in monolayters for many generations may not represent the real tumor structure and components. Some researchers have proposed more experimentation with orthotopic tumor models to elucidate which organ and disease are suitable for this promising treatment strategy (79).

The size of mice becomes another disadvantage for investigation of local therapies and drug delivery, due to the disparities in scale. On small animal cancer models, the tumors being treated by FT are usually small (a few millimeters) and spherical. Clinical devices, designed to treat tumors at centimeter scale, are usually too big for the tumors on mice, so customized devices have to be used for preclinical research. Monoclonal antibodies are each unique, so ICI drugs for mice are inherently different than for humans, making it difficult to translate the treatment effect from mouse to human. Scaling up the setup and dose of FTs and ICIs remains a big challenge. The size difference may also have an impact on the ICI timing and dosing.

The difference in drug delivery methods between animals and humans also becomes a challenge in translational research. ICI drugs are typically infused intravenously in clinical studies while different drug delivery methods, such as intraperitoneal and intratumoral injection, are typically used in animal studies. Nonetheless, studies of syngeneic cell lines or genetically engineered mouse models have supported progress to date on cancer immunotherapy and that situation is not likely to change in the near future.

## 9 Future directions for combination therapy of FT with ICI

**Figure 4:**
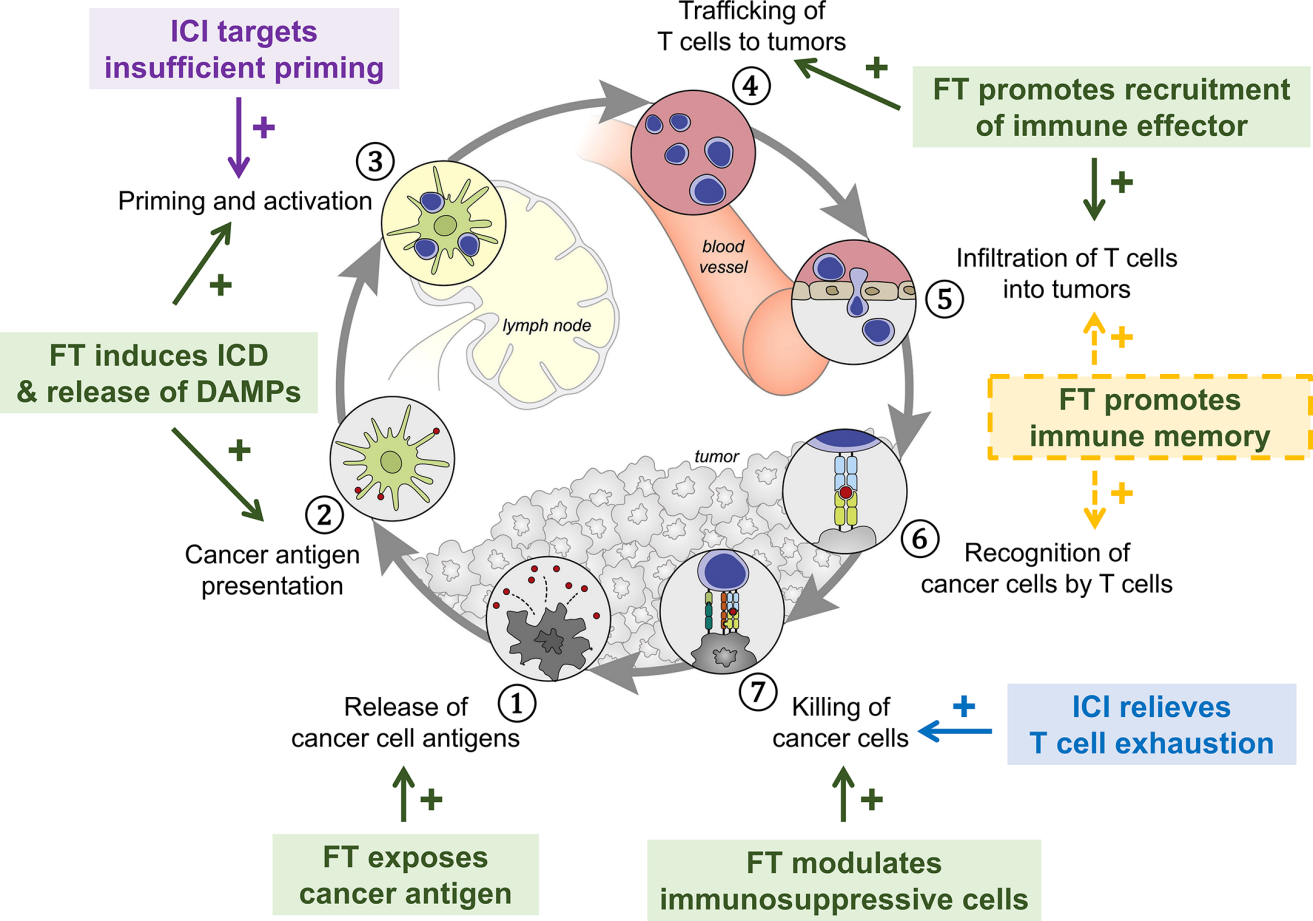
The intervention of focal therapy (FT) and immune checkpoint inhibitor (ICI) in the cancer-immunity cycle. The cycle has several major steps that have both immune-stimulatory and inhibitory factors. The immune response to the FT can be harnessed to amplify and broaden T cell responses while the ICI can target the immune regulatory feedback mechanisms that halt the development or limit the immunity. Working together, FT and ICI can facilitate the maintenance of central, effector and tissue-resident memory CD8 T cells to promote long-term immunosurveillance and protection. ICD, immunogenic cell death; DAMPs, danger-associated molecular patterns. Figure adapted from original figure by Chen et al. (316) with permission.

T cell responses that recognize and eradicate cancer cells are an important part of the cancer-immunity cycle (316), as shown in **Figure 4**. The generation of immunity to cancer is a cyclical process that can be self-propagating, leading to complete eradication of cancer. The cycle has several major steps that have both immune-stimulatory and inhibitory factors. In order to keep its positive cyclic process progressing, the goal of the therapeutic strategy is to ensure the passage along the steps in the cancer-immunity cycle are free from checkpoint(s). Therefore, the strategy with FT and ICI is the modulation of the cancer-immunity cycle to improve immediate- or long-term response. To accomplish this, the most effective approach is to selectively target the step or steps where the cycle is blocked or checked.

To design an effective approach involving FT and ICI, the following 3 steps are needed. First, the cancer immune microenvironment must be assessed to identity the “bottlenecks” of the cycle. Second, with the knowledge of the limiting steps, the prescription of FT and/or ICI needs to target the stimulatory and inhibitory factors within each step, without introducing additional bottlenecks to the system. Finally, auxiliary immunotherapy and adjuvant therapy can be prescribed to better address the bottlenecks and stimulate antitumor immunity.

### 9.1 Identifying the bottlenecks

For a tumor to exist or even to progress, the crucial steps of the cancer-immunity cycles leading to cancer elimination must be arrested. To determine the bottlenecks, the tumor immune microenvironment must be fully assessed before the combination therapy, as the microenvironment of cancer varies among tumors and patients. Predictive biomarkers that are present at baseline prior to treatment initiation and predict ICI response can be evidence of stimulatory signal or inhibitory/suppressive factors. Such factors include the genetics of the cancer cells that dictate the mutation burden and the attributes (i.e., population, phenotype, and the location) of immune cells.

Equally important are the preemptive biomarkers, which are generated upon treatment initiation. Biomarkers associated with good prognoses can be evidence of effective treatment that alleviates the bottlenecks in the cancer-immunity cycle. Biomarkers contrary to prediction can be signs of failed strategy and will require changes of strategy. One challenge, however, is that some biomarkers cannot be detected in peripheral blood and will require tumor biopsy. Other challenges involve elusive data from systematic comparisons of the immunogenic effects of different modalities of focal therapy. The immune cell populations (both the immune effectors and regulators) and immunological features following FT, as monotherapy or with ICI, in addition to their comparisons to conventional treatment, remain largely unknown.

### 9.2 Prescribing the immunomodulation

The summary of potential solutions to the bottlenecks is presented in **Table 12**. Optimally, limiting step(s) of a given cancer condition should be identified before prescribing the potential solution that specifically targets each step. Designing the FT+ICI strategy to achieve the listed solutions is crucial for the success of the cancer therapy. Auxiliary therapy (e.g., other forms of immunotherapy, anti-angiogenic therapy) can be used to augment the immunomodulatory effects by FT and/or ICI to alleviate the limits on each of the seven steps.

**Table 12:** Potential solutions to the bottlenecks in the major steps that arrest the self-propagating cancer-immunity cycle by combinatory focal therapy (FT) and immune checkpoint inhibitor (ICI).

Bottlenecks in cancer-immunity cycle	Potential solution by FT and ICI	Potential auxiliary therapy	Reference of examples
(1) **Release of cancer cell antigens**	• FT resulting in more immunogenic or necrotic cell death • FT resulting in less tolergenic or apoptotic cell death		(317)
(2) **Cancer antigen presentation**	• FT promoting DC maturation and activation • FT releasing proinflammatory cytokines (e.g., TNF-α, IL1, IFN-α) • FT reduces inhibitory cytokines (e.g., IL-10, IL-4, IL-13) • FT releasing endogenous adjuvants (e.g., CDNs, ATP, HMGB1)	Cancer vaccines IFN-α GM-CSF Anti-CD40 TLR agonists	(318, 319)
(3) **Priming and activation**	• FT releasing more stimulatory factor (e.g., IL-2, IL-12) ▪ ICI targeting inhibitory pathway of CD8 T cell activation (e.g., anti-CTLA-4) ▪ ICI targeting inhibitory pathway of Th1 CD4 T cell activation ▪ ICI reducing Treg function	Anti-CD137 Anti-OX40 Anti-CD27 IL-2 IL-12 Prostaglandin inhibitors	(318, 320–322)
(4) **Trafficking of T cells to tumors**	• FT promoting T cells trafficking	CX3CL1 CXCL9 CXCL10 CCL5	(320, 323)
(5) **Infiltration of T cells into tumors**	• FT promoting T cells infiltration • FT^#^ contributing to the establishment of immune memory	Anti-VEGF ETRAs	(324)
(6) **Recognition of cancer cells by T cells**	• FT^#^ contributing to the establishment of immune memory	• FT that upregulates peptide-MHC expression on cancer cells CARs	(319)
(7) **Killing of cancer cells**	• FT that suppresses Treg, MDSC and M2 macrophage ▪ ICI targeting CD8 T cell exhaustion (e.g., anti-PD-1, anti-TIM-3, anti-LAG-3) ▪ ICI targeting the source of inhibitory signaling to CD8 T cells (e.g., anti-PD-L1, anti-PD-L2 etc.)	IDO inhibitors • FT that relieves hypoxia	(319, 325–327)

CDNs, cyclic dinucleotides; VEGF, vascular endothelial growth factor; ETRAs, endothelin receptor antagonists; CARs, chimeric antigen receptors. ^#^Long-term effect, not immediate reaction.

## Author contributions

MJ, SF, and QS contribute to the conception, MJ and QS were involved in acquisition, analysis, or interpretation of data; MJ, SF, and QS drafted the work and/or substantively revised it. All authors contributed to the article and approved the submitted version.
