# Supplementary material for: Combining energy-based focal ablation and immune checkpoint inhibitors: preclinical research and clinical trials
Source: Front Oncol. 2023 May 1;13:1153066. doi: 10.3389/fonc.2023.1153066 (PMC10211342; doi:10.3389/fonc.2023.1153066)
Supplement: Supplementary file 1 [file Table_1.docx]

Supporting information:

Combining Energy-based Focal Ablation and Immune Checkpoint Inhibitors: Preclinical Research and Clinical Trials

Contents

[Supplemental Table S1. Literature research for preclinical FT + ICI 2](#_Toc125058417)

[Supplemental Table S2. Immunological assessment of FT and ICI in preclinical models. 5](#_Toc125058418)

[Supplemental Table S3. NP-mediated hyperthermia therapy + ICI in vivo research on animals 8](#_Toc125058419)

[Supplemental Table S4. Literature research for clinical FT + ICI 9](#_Toc125058420)

[Supplemental Table S5. 5-year survival rate and TMB by cancer type. 10](#_Toc125058421)

[Supplemental Table S6. Results of FT+ICI clinical studies for cancers 14](#_Toc125058422)

| Focal therapy keywords | Ablation, radiofrequency, microwave, thermal, hyperthermia, cryoablation, cryotherapy, cryosurgery, electroporation, irreversible electroporation, pulsed electric field, high-intensity focused ultrasound, laser, photothermal, histotripsy, magnetic fluid hyperthermia, magnetic thermal therapy |
| --- | --- |
| Immunotherapy keywords | Immunotherapy, immunomodulation, immune checkpoint |
| Database | PubMed  Web of Science  Google Scholar |
| Exclusion | Conference presentation, conference proceeding or abstract  Preprint  Book chapters  No in vivo tumor treatment  Commentary, opinion, or review  In languages other than English |

# Supplemental Table S1. Literature research for preclinical FT + ICI

| **FT + ICI** | **Dose and timing** | **Immunomodulatory assessment** | | | | | **Ref** |
| --- | --- | --- | --- | --- | --- | --- | --- |
|  |  | **DCs** | **CD8+ T cells** | **CD4+ T cells** | **Immuno-suppressive cells** | **Cytokines &**  **Small molecules** |  |
| RFA + Anti-CTLA-4 | IP, 200 µg on days 0, 3 and 6 | ― | spleen + LN | ― | ― | ― | Den 2004^1^ |
| RFA + Anti-CTLA-4 | IP, 200 µg on days 0, 3 and 6 | LN | LN + spleen | ― | Treg depletion | ― | Den 2006^2^ |
| RFA + Anti-PD-1 | IP, 200 μg on days 1, 4, 7 and 10 | ― | IT + TDLN + spleen | IT | Treg | TNF-α, IFN-γ | Shi 2016^3^ |
| RFA + Anti-PD-1 | IP, 200 μg on days 1, 4, 7 and 10 | ― | IT + spleen | IT | Treg + MDSC | TNF-α, IL-10, TGF-β, CCL2 | Shi 2019^4^ |
| RFA + Anti-CTLA-4 | IV, 10 µg on days 1, 3, 5 and 7 | ― | IT + spleen | IT | Treg + MDSC | TNF-α, IFN-γ | Han 2019^5^ |
| RFA + Anti-CTLA-4 | IP, 200 μg on days 1, 3, and 5 | ― | IT | IT | ― | ― | L. Zhang 2020^6^ |
| RFA + Anti-CTLA-4 | IP, 200 μg on days 1, 3, and 5 | ― | IT | IT | ― | ― | L. Zhang 2020^7^ |
| MWA + Anti-CTLA-4 | IP, 200 μg on days 3, 10 and 17 | ― | spleen | spleen | ― | ― | Chen 2009^8^ |
| MWA + Anti-PD-1 +  Anti-CTLA-4 | Anti-PD-1: IP, 40 μg on days 0, 3, and 6  Anti-CTLA-4: IP, 200 μg on days 0, 3, and 6 | ― | peripheral + IT | ― | Treg + MDSC | IFN-γ | Zhu 2018^9^ |
| MWA + Anti-PD-1 +  Anti-CTLA-4 | Anti-PD-1: IP, 40 μg on days 0, 3, and 6  Anti-CTLA-4: IP, 200 μg on days 0, 3, and 6 | ― | IT + spleen | IT + spleen | ― | IFN-γ, IL-18, IL-2, IL-4, IL-10 | Duan 2020^10^ |
| MWA + Anti-PD-1 | IP, 200 μg on days 3, 5, 7 and 9 | ― | IT | IT | Treg | IFN-γ, TNF-α | Huang 2022^11^ |
| MWA + Anti-LAG3 | IP, 200 μg on days 1, 4, 7 and 10 | IT | IT | IT | Treg | IFN-γ, TNF-α | Shao 2022^12^ |
| HIFU + Anti-PD-1 | IP, 200 μg on days 0 and 7 | ― | IT + LN + spleen | IT + LN + spleen | Treg + MDSC | ― | Silvestrini 2017^13^ |
| HIFU + Anti-PD-1 | IP, 200 μg on days -10, -3 and 3 | IT | IT | IT | ― | IFN-α, IFN-β | Chavez 2018^14^ |
| HIFU + Anti-CTLA-4 | IV, 10 µg on days 41, 43, 45 and 47 (HIFU)  IV, 10 µg on days 1, 3, 5 and 7 (RFA+HIFU) | ― | IT + spleen | IT | Treg + MDSC | TNF-α, IFN-γ | Han 2019^5^ |
| HIFU + Anti-PD-1 | IP, 200 µg on days -7, -4, -1, 2 and 5  or on days 7, 10, 13, 16 and 19 | ― | IT | ― | ― | ― | Sheybani 2020^15^ |
| HIFU + Anti-PD-1 | IP, 200 μg on days -9, -2, and 4 | IT | IT | IT | Treg + MDSC | IL-6, IL-1β, CXCL9, CXCL10 | Fite 2021^16^ |
| HIFU + Anti-CTLA-4 + Anti-PD-1 | IP, 200 μg on days -3, 0, 3, 6, 9, 12, 15 and 18 | ― | IT + TDLN + spleen | IT + TDLN + spleen | Treg + MDSC | TNF-α, IFN-γ | Mouratidis 2021^17^ |
| CRYO or RFA + Anti-CTLA-4 | IP, 200 µg on days 0, 3 and 6 | LN | LN + spleen | ― | Treg depletion | ― | Den 2006^18^ |
| CRYO + Anti-CTLA-4 | IP, 200 μg on day 1, 100 μg on days 4, 7, and 10 | ― | IT + spleen,  CD8 depletion | IT | Treg | ― | Waitz 2012^19^ |
| CRYO + Anti-CTLA-4 | IP, 100 μg after ablation on day 0 | ― | IT + TDLN + spleen | IT + TDLN + spleen | Treg | IL-2, TGF-β | Li 2014^20^ |
| CRYO + Anti-CTLA-4 | IP, 200 μg on days 1 and 4 | ― | spleen | spleen | ― | ― | Kudo-Saito 2016^21^ |
| CRYO + Anti-PD-1 or Anti-CTLA-4 | Anti-CTLA-4: IP, 20 μg on days -2, 0, 2, and 4  Anti-PD-1: IP, 200 μg on days -2, 0, 2, and 4 | ― | IT + TDLN + spleen  CD8 or CD3 depletion | IT + TDLN + spleen | Treg | IFN-γ, TNF-α, IL-2, | Benzon 2018^22^ |
| CRYO + Anti-PD-1 | IP, 200 μg on days 1, 4, 7 and 10 | ― | IT | ― | ― | IFN-γ, IL-10 | Zhu 2019^23^ |
| CRYO + Anti-PD-1 | IP, 20 mg/kg on day 0, 10 mg/kg on days 5 and 10 | ― | IT | IT | Treg | ― | Annen 2022^24^ |
| CRYO + Anti-PD-L1 | IP, 10 mg/kg every 3 days after CRYO | IT | IT | ― | MDSC, TAM | IFN-γ, CXCL9 | Tan 2022^25^ |
| CRYO + Anti-PD-1 | IP, 200 μg on days 1, 4, 7 and 10 | ― | IT | IT | Treg + MDSC | IL-12, IFN-γ | Yu 2023^26^ |
| IRE + Anti-PD-1 | IP, 200 μg on days -1, 1 and 3 | IT | IT | IT | Treg + MDSC | ― | Narayanan 2019^27^ |
| IRE + Anti-PD-1 | IP, 100 μg on days 0, 2, 4, 6, 8 and 10 | IT | IT + spleen,  CD8 depletion | IT + spleen | Treg + MDSC | ― | Zhao 2019^28^ |
| IRE + Anti-PD-L1 | IP, 80 μg on days 3, 6 and 9 | ― | IT | ― | ― | ― | Qian 2020^29^ |
| IRE + Anti- CTLA-4 | IP, 200 μg on day 1, 100 μg on days 4, 7 and 10 | ― | Peripheral blood & IT | Peripheral blood & IT | Treg | ― | Burbach 2021^30^ |
| IRE + Anti-PD-L1 | IP, 200 μg on days -1, 1, 3 and 5 | IT | IT + TDLN,  CD8 depletion | IT | Treg + MDSC | IL-2, IFN-γ, and TGF-β | Babikr 2021^31^ |
| IRE + Anti-PD-L1 | IP, 200 μg every 3 days up to four weeks following IRE | ― | IT | ― | MDSC | ― | Shi 2021^32^ |
| USMB + Anti-PD-1 | IP, 200 μg on days 0, 3, 6, 9 and 12 | ― | IT | IT | Treg | IFN-γ | Bulner 2019^33^ |
| Histotripsy + Anti-CTLA-4 + Anti-PD-L1 | IP, 100 µg on days 1, 4 and 7 | IT +  TDLN | IT + LN + spleen | IT + LN + spleen | Treg | IFN-γ, IL-10, IL-6, GM-CSF | Eranki 2020^34^ |
| Histotripsy + Anti-PD-1 | IP, 200 μg on days 3, 5, 7 | ― | IT | ― | MDSC | ― | Nam 2020^35^ |
| Histotripsy + Anti-CTLA-4 | IP, 200 μg on days -1, 2, 3 (B16GP33)  or days -7, -4, -1, 2 (Hepa1-6) | ― | IT | ― | ― | ― | Qu 2020^36^ |
| M-HIFU + Anti-PD-L1 | IP, 100 μg on days 5, 8, 11 | IT | IT | IT | Treg | TGF-β, IFN-γ, TNF-α | Abe 2022^37^ |
| US irradiation + Anti-PD-1 | IP, 250 μg every 4 days for  a total of 5 times | ― | ― | ― | ― | ― | Hayashi 2022^38^ |
| Hyperthermia + Anti-CTLA-4 | IP, 150 μg on days -3, 0, 3 | ― | ― | ― | ― | ― | Ibuki 2021^39^ |

# Supplemental Table S2. Immunological assessment of FT and ICI in preclinical models.

*day 0 is the day when focal therapy was applied.

| **Cancer model** | **Material** | **Energy source** | **ICI** | **Ref** |
| --- | --- | --- | --- | --- |
| **NP-mediated photothermal therapy** | | | | |
| 4T1 | SWNTs | 808nm NIR laser | Anti-CTLA-4 | Wang 2014^40^ |
| CT26, 4T1 | PLGA-ICG-R837 NPs | 808nm NIR laser | Anti-CTLA-4 | Chen 2016^41^ |
| Neuro2a (neuroblastoma) | Prussian blue NPs | 808nm NIR laser | Anti-CTLA-4 | Cano-Mejia 2017^42^ |
| MB49 (bladder cancer) | Gold nanostars | 808nm NIR laser | Anti-PD-L1 | Liu 2017^43^ |
| 4T1 | Fe3O4-R837 SPs | 808nm NIR laser | Anti-PD-L1 | Ge 2018^44^ |
| CT26, 4T1 | APP- and HAuNS-loaded PLGA NPs | 808nm NIR laser | Anti-PD-1 | Luo 2018^45^ |
| B16-F10 | mPEG-GNRs@BSA/R837 nanocomplexes | 1064nm NIR laser | Anti-PD-1 | Zhou 2018^46^ |
| 4T1 and EMT6 (breast cancer) | personalized cancer vaccine (PVAX) | 808nm NIR laser | Anti-PD-L1 | Wang 2018^47^ |
| U14 (cervix cancer) | PEGylated CMS@GOx | 1064 nm NIR laser | Anti-CTLA-4 | Chang 2019^48^ |
| 4T1 | FA-CD@PP-CpG | 808nm NIR laser | Anti-PD-L1 | L. Chen 2019^49^ |
| B16-F10 | pDA-VNP | 808nm NIR laser | Anti-PD-1 | W. Chen 2019^50^ |
| 4T1 | PLGA-ICG NPs | 808nm NIR laser | Anti-PD-L1 | Han 2019^51^ |
| CT26 | RBC–MoSe2 nanosheets | 808nm NIR laser | Anti-PD-1 | He 2019^52^ |
| 4T1 | SWNT-GC | 1064nm NIR laser | Anti-CTLA-4 | Li 2019^53^ |
| 4T1 | BPQD-RM nanovesicle | 808nm NIR laser | Anti-PD-1 | Liang 2019^54^ |
| HCC827 (lung cancer) | GNPs@PSS/PDADMAC | 633nm laser | PD-L1 siRNA | Liu 2019^55^ |
| 4T1 | PDA-PEG-R848-CD NPs | 808nm NIR laser | Anti-PD-L1 | Lu 2019^56^ |
| 4T1-Luc | DTX-IR820-CF27 NPs | 808nm NIR laser | Anti-PD-L1 | Peng 2019^57^ |
| 4T1 | Bi2Se3 NC-PEG/R848 | 808nm NIR laser | Anti-PD-L1 | Song 2019^58^ |
| 4T1 | PPy@CPT-HA-IRDye800CW NPs | 808nm NIR laser | Anti-PD-L1 | Sun 2019^59^ |
| 4T1 | Polydopamine NPs | 808nm NIR laser | Anti-PD-L1 | Tian 2019^60^ |
| 4T1 | CuS NPs-PEG-Mal | 808nm NIR laser | Anti-PD-L1 | Wang 2019^61^ |
| 4T1 | Cu2-xTe NPs | NIR-II, 1000–1350 nm | Anti-PD-1 | M. Wen 2019^62^ |
| CT26 | Pd-Dox@TGMs NPs | 808nm NIR laser | Anti-PD-L1 | Y. Wen 2019^63^ |
| CT26 | IDOi/rGO nanosheets | 808nm NIR laser | Anti-PD-L1 | M. Yan 2019^64^ |
| 4T1 | PDA@UCNP-PEG/Ce6 | 980nm NIR laser | Anti-PD-1 | S. Yan 2019^65^ |
| 4T1 | Au@Pt NPs | 808nm NIR laser | Anti-PD-L1 | Yang 2019^66^ |
| 4T1-luc, B16-F10-luc | Gel-BPQD-CCNVs | 808 NIR laser | Anti-PD-1 | Ye 2019^67^ |
| 4T1 | FePt/MoS2-FA nanocomposites | 808nm NIR laser | Anti-CTLA-4 | D. Zhang 2019^68^ |
| B16-F10-luc | GOP@aPD1 NPs | 660nm laser | Anti-PD-1 | N. Zhang 2019^69^ |
| Neuro2a (neuroblastoma) | CpG-PBNPs | 808nm NIR laser | Anti-CTLA-4 | Cano-Mejia 2020^70^ |
| B16-OVA, ID8 (ovarian cancer) | PEG-CuS NPs | 1064nm PW laser | Anti-PD-1 | Cao 2020^71^ |
| 4T1 | IONPs | 885nm NIR laser | Anti-CTLA-4 | Chen 2020^72^ |
| fluc-4T1 | WO2.9-WSe2-PEG NPs | 808nm NIR laser | Anti-PD-L1 | Dong 2020^73^ |
| 4T1 | BG@NbSiR scaﬀold | 808nm NIR laser | Anti-PD-L1 | He 2020^74^ |
| CT26 | Cu-PPT NPs | 650 + 808nm laser | Anti-PD-L1 | Hu 2020^75^ |
| MC38 | ICG-liposome NPs | 808nm NIR laser | Anti-PD-1 | Huang 2020^76^ |
| B16-F10 | AuMNs nanosheets | 808nm NIR laser | Anti-PD-L1 | Li 2020^77^ |
| B16-F10 | mPEGPep-IDOi NPs | 808nm NIR laser | Anti-PD-L1 | Liu 2020^78^ |
| 4T1 | BP/TPP/RGD/R848 NPs | 808nm NIR laser | Anti-CTLA-4 | Zeng 2020^79^ |
| 4T1 | γ-PGA@GOx@Mn,Cu-CDs | 730nm laser | Anti-PD-L1 | M. Zhang 2020^80^ |
| 4T1 | Ap-AuND | NIR-II, 1000−1700 nm | Anti-PD-L1 | Y. Zhang 2020^81^ |
| K7 (osteosarcoma) | GNR-PEI/cGAMP NPs | 808nm NIR laser | Anti-PD-L1 | Zhao 2020^82^ |
| CT26 | COF@ICG@OVA NPs | 650nm + 808nm laser | Anti-PD-L1 | Zhou 2020^83^ |
| CT26 | FePSe3@APP@CCM | 808nm NIR laser | Anti-PD-1 | Fang 2021^84^ |
| B16-F10 | AuNR/DOX gel | 808nm NIR laser | Anti-PD-L1 | Feng 2021^85^ |
| CT26 | A/Au@MSMs-P | 808nm NIR laser | Anti-PD-1 | Gao 2021^86^ |
| 4T1 | BG@NbSiR | 808nm NIR laser | Anti-PD-L1 | He 2021^87^ |
| 4T1 | PLGA-pTA NPs | 808nm NIR laser | Anti-PD-L1 | Huang 2021^88^ |
| 4T1 | Au/Ag NRs | 1064nm laser | Anti-CTLA-4 | Jin 2021^89^ |
| B16-OVA | FKF-OVAp@BP | 808nm NIR laser | Anti-PD-L1 | Li 2021^90^ |
| Panc02-H7 | Fe2P-PEG-Mal NRs | 980nm laser | Anti-PD-L1 | S. Liu 2021^91^ |
| SMM103 | ASP∩V | 808nm NIR laser | Anti-PD-1 | X. Liu 2021^92^ |
| MB49 (bladder cancer) | GNSs | 808nm NIR laser | Anti-PD-L1 | Y. Liu 2021^93^ |
| EMT6 (breast cancer) | SWCNTs | 980nm NIR laser | Anti-CTLA-4 | McKernan 2021^94^ |
| CT26 | APTEDB-cyNP@CpG | 808nm NIR laser | Anti-PD-1 | Noh 2021^95^ |
| 4T1 | IR780/DPPC/BMS | 808nm NIR laser | Anti-PD-L1 | Tian 2021^96^ |
| 4T1 | CuCo(O)/GOx@ PCNs | 808nm NIR laser | Anti-PD-1 | Q. Wang 2021^97^ |
| CT26 | GNC-Gal@CMaP nanocomposites | 808nm NIR laser | Anti-PD-L1 | S. Wang 2021^98^ |
| B16 | ICG-NP MNs | 808nm NIR laser | Anti-PD-L1 | Yang 2021^99^ |
| CT26 | FOIB@Lip | 808nm NIR laser | Anti-PD-L1 | J. Yu 2021^100^ |
| Pan 02 | HSA-BMS@CAP-ILTSL | 808nm NIR laser | Anti-PD-L1 | Q. Yu 2021^101^ |
| 4T1 | BBPQDs | 808nm NIR laser | Anti-PD-L1 | P. Zhao 2021^102^ |
| 4T1 | ZIF-PQ-PDA-AUN | 808nm NIR laser | Anti-PD-1 | W. Zhao 2021^103^ |
| K7 (osteosarcoma) | GNR-PEI/cGAMP complex | 808nm NIR laser | Anti-PD-1 | X. Zhao 2021^104^ |
| 4T1 | AuNR@BSA/MnO2 | 808nm NIR laser | Anti-PD-1 | M. Zhou 2021^105^ |
| 4T1 | PPor NPs | 808nm NIR laser | Anti-PD-1 | Cao 2022^106^ |
| 4T1 | TTF-F4TCNQ | 1064nm laser | Anti-PD-1 | Chen 2022^107^ |
| H22 | SPLI | 1064nm laser | Anti-PD-1 | Du 2022^108^ |
| 4T1 | IONPs | 808nm NIR laser | Anti-PD-L1 | Gao 2022^109^ |
| LLC (lung carcinoma) | GNRs-PEI/cGAMP NPs | 808nm NIR laser | Anti-PD-L1 | Jin 2022^110^ |
| 4T1 | CHINPs | 808nm NIR laser | Anti-PD-1 | Lin 2022^111^ |
| CT26 | OIMH NPs | 808nm NIR laser | Anti-PD-L1 | Liu 2022^112^ |
| H22 | CCF‑LDHs | 808nm NIR laser | Anti-PD-L1 | Lu 2022^113^ |
| CT26 | ALG | 1064nm laser | Anti-PD-L1 | Ning 2022^114^ |
| 4T1 | LGT | 1064nm laser | Anti-CTLA-4 | Pu 2022^115^ |
| 4T1 | FeWOx-PEG | 1060nm laser | Anti-PD-L1 | Xiang 2022^116^ |
| 4T1 | TPA-BT-DPTQ | 808nm NIR laser | Anti-PD-L1 | Yan 2022^117^ |
| MCF-7 | END-PANi-PVP@R837 NPs | 808nm NIR laser | Anti-PD-L1 | Yasothamani 2022^118^ |
| CT26 | aNP@IR780 | 808nm NIR laser | Anti-PD-L1 | Yu 2022^119^ |
| 4T1 | AIPH@MS-CTPP | 1064nm laser | Anti-PD-1 | Zhang 2022^120^ |
| **NP-mediated magnetic hyperthymia** | | | | |
| 4T1-luc | MION | 155kHz AMF | Anti-PD-1 + Anti CTLA-4 | Oei 2019^121^ |
| CT26 | FeNPs | 100kHz AMF | Anti-CTLA-4 | Chao 2019^122^ |
| 4T1 | FVIOs | 365kHz AMF | Anti-PD-L1 | Liu 2019^123^ |
| 4T1 | M-MONs@Ce6 | 660nm laser &262 kHz ACMF | Anti-CTLA-4 | Wang 2019^124^ |
| 4T1 | CoFe2O4@MnFe2O4 NPs | 577 kHz AMF | Anti-PD-L1 | Pan 2020^125^ |

# Supplemental Table S3. NP-mediated hyperthermia therapy + ICI in vivo research on animals

| Focal therapy keywords | Ablation, radiofrequency, microwave, thermal, thermal therapy, hyperthermia, cryoablation, cryotherapy, cryosurgery, electroporation, irreversible electroporation, pulsed electric field, high-intensity focused ultrasound, laser, laser interstitial thermotherapy |
| --- | --- |
| Immunotherapy keywords | Immunotherapy, immunomodulation, immune checkpoint, CTLA-4, anti-CTLA-4, PD-1, anti-PD-1, PD-L1, anti-PD-L1, avelumab, pembrolizumab, atezolizumab, durvalumab, nivolumab, cemiplimab, ipilimumab, tremelimumab |
| Database | ClinicalTrials.gov  PubMed  Web of Science  Google Scholar |
| Exclusion | Only monotherapy in treatment plan (combination therapy is not part of study design)  No clinical trial identifier  Terminated or withdrawn before completion  Conference presentation or abstract  Preprint  Commentary, opinion, or review |

# Supplemental Table S4. Literature research for clinical FT + ICI

| Cancer type | 5-year survival rate | Tumor Mutation Burden^#^ (mutations/Mb)^126^ |
| --- | --- | --- |
| glioma | 6.8^127^ | 2.654 |
| pancreatic | 11^128^ | 2.113 |
| BTC | 10^129^ | 2.5 |
| lung cancer | 22^130^ | 7.135 |
| HCC | 20^131^ | 3.6 |
| colorectal cancer | 65^132^ | 4.486 |
| RCC | 76^133^ | 2.7 |
| lymphoma | 73^134,135^ | 7.716 |
| breast cancer | 90^136^ | 3.636 |
| melanoma | 93^137^ | 13.318 |
| prostate cancer | 98^138^ | 2.804 |
| Basal Cell Carcinoma | 100^139^ | 47.3 |
| Soft Tissue Sarcoma | 65^140^ | 2.247 |
| Urothelial carcinoma | 77^141^ | 6.0768 |
| Mesothelioma | 12^142^ | 1.8 |

# Supplemental Table S5. 5-year survival rate and TMB by cancer type.

^#^When different cancer subtypes are involved, the TMB for a general disease is the weighted average of TMBs for different subtypes based on sample size.

| **Identifier** | **Improve Therapeutic effect** | **Enhance Immune response** | **Reduce adverse events** | **Ref** |
| --- | --- | --- | --- | --- |
| NCT03873818 | Objective response rates (ORR) of cryoablated lesions were 75% (9/12), ORR of distant lesions were 40%  (4/10) and total ORR (local+distant) were 50% (ipilimumab-63%  (5/8), pembrolizumab-25% (1/4)). Local disease control rates (DCR) were 83%, distant DCR were 60%, and overall DCR were 75%. The progression-free survival rate at  6 months was 57%. | No data | Grade 3 irAE toxicities observed in 2 patients including colitis and hypophysitis. No other grade ¾ irAEs or toxicities related to cryo. | ^143^ |
| NCT03290677 | ORR was 18% and DCR was 47% (3 PR, 5 SD) | No data | No de novo immune-related adverse events post-ablation and no grade 4/5 events. | ^144^ |
| NCT02833233 | Ipi/nivo/cryo was well tolerated overall | Greater T cell activation on peripheral T cells, Treg, downregulation | One pt on an aromatase inhibitor had grade 4 liver toxicity, One pt developed grade 1 hyperthyroidism | ^145^ |
| NCT02821754 | Median PFS was 2.1m and median OS was 5.6 m. DCR was 45% (SD). Median OS and PFS was similar in the group that received combination therapy vs immunotherapy alone with a median OS of 6.8 m vs 6.7 m and 2.0 m vs 2.7 m respectively. | No data | The most common grade 3- 4 adverse events were lymphopenia (27%), increased AST (41%), increased alkaline phosphatase (32%) and elevated bilirubin (27%) | ^146^ |
| NCT02626130 | Median OS: 22.7 months for cryo-tremelimumab combination therapy group and 33.7 months for tremelimumab monotherapy group.  PFS: 3.0 months for combination therapy group and 5.0 months for tremelimumab-only group. | Treatment increases immune cell infiltration and tertiary  lymphoid structures in clear cell but not in non-clear cell. In clear cell, cryoablation plus  tremelimumab leads to a significant increase in immune cell infiltration. | ≥ grade 3 treatment-related adverse events in 16 of 29 patients (55%) including 6 diarrhea/colitis, 3 hepatitis, 1 pneumonitis, and 1 glomerulonephritis. Toxicity leading to treatment discontinuation occurs in 5 patients in each arm. | ^147^ |
| NCT02489357 | 42% of patients had a PSAs of <0.6ng/mL at one year though only 2 of these patients had recovered their testosterone at this time point. Median progression-free survival was 14 months, and median systemic therapy-free survival was 17.5 months. | PD-L1 staining was absent on tumor cells post treatment. Clonality and Daley-Smith richness were relatively stable at baseline and following 6 cycles of pembrolizumab overall for all patients. 1/10 patient appeared to have a higher frequency of contracted and expanded T cell clonotypes following 6 cycles of pembrolizumab. | All adverse events were grade ≤2, and no apparent complications from cryotherapy. | ^148^ |
| NCT02423928 | 6 patients partial response or stable disease. Median PFS was 150 days in total cohort. | Higher number of novel TCR clonotypes in men with non-progressive disease | AE profile of the total cohort (n=18) was comparable with interim reports (n=13); of 20 possible DC-related AEs one was severe (urinary retention) and 19 mild-to-moderate, and spread independent of treatment regimen | ^149^ |
| NCT01502592 | Pre-op cryo and ipi, alone or in combination, are safe/tolerable with no delays in pre-planned surgery. Tumor necrosis/infarction was observed in 9/12 pts who underwent cryo. | Sustained peripheral elevations in activated (ICOS+) and proliferating (Ki67+) CD8+ T cells, and post-treatment proliferative T-effector cells. sustained peripheral elevations, sustained peripheral elevations in: Th1-type cytokines | One pt had Grade III toxicity (unrelated rash after ipilimumab) | ^150^ |
| NCT03939975 | Of all 50 patients treated with anti-PD-1 therapy, the rate of response, stable  disease, atypical and typical progression were 10%, 42%, 32%,  and 12%, respectively. Additional ablation increased the response rate from 10 to 24%. The median time to  progression, progression-free survival, and overall survival was 6.1 months, 5 months, and 16.9 months, respectively | No data | At least one anti-PD-1  inhibitor-related toxicity has occurred in 41 of the 50  patients and, among those, 7 were as serious AEs. No ablation related severe complications (Grade E) or death (Grade F) were recorded. | ^151^ |
| NCT03864211 | The objective response rate (ORR) was 18.8% in ICI alone group, 37.5% in combination group. In combination group, 2 patients (12.5%) achieved complete response. | No data | 75% patients experienced treatment-related adverse events (TRAE). The most common TREAs were mild (grade 1/2). Grade 3/4 TRAEs was reported in 18.7% of patients in ICI group, 25.0% in combination group. No TRAE led to death. | ^152^ |
| NCT02821754 | The average overall survival in the 58 pts was 10.1 months; 41.6% were alive at 12 months and 5.5 % were alive at 61 months. A select group of patients had exceptional overall survivals up to 67 months. | No data | No new late toxic effects were noted during follow up. | ^153^ |
| NCT02437071 | Interim ORR is 9% (1 of 11) in the RT cohort. | No data | Grade 1 or 2 drug-related AEs were reported in 73% of pts, the most frequent were fatigue (23%), rash (15%), and nausea (15%). | ^154^ |
| NCT01853618 | 2 (12.5%) patients achieved a confirmed partial response and 5 patients (31.3%) achieved stable disease. Median progression free survival (PFS) and overall survival (OS) were 3.4 months and 6.0 months, respectively. | Increased circulating activated human leukocyte antigen, DR isotype ([HLA-DR] positive) CD8+ T cells. Tremelimumab expanded TCR repertoire, but not reaching statistical significance. | No dose-limiting toxicities were encountered. The common treatment-related AEs included lymphopenia, diarrhea, and elevated transaminases. | ^155^ |
| NCT01853618 | Confirmed partial response (n=5). Marked reduction in viral load (n=12). Six and 12-month probabilities of tumor progression free survival for this refractory HCC population were 57.1% and 33.1% respectively, with median time to tumor progression of 7.4 months, Median overall survival was 12.3 months | Tremelimumab therapy increased CD4+-HLA-DR+, CD4+PD-1+, CD8+HLA-DR+, CD8+PD-1+, CD4+ICOS+ and CD8+ICOS+ T cells in the peripheral blood. PD-1 expression was increased on alpha fetal protein (AFP) and surviving-specific CD8 T cells upon tremelimumab treatment. An increase of tumor infiltrating CD3+ T cells  were also seen in these patients. One cycle of tremelimumab significantly decreased peripheral clonality, while no additional effects were seen after loco-regional therapy. | No dose-limiting toxicities, the most common toxicity was pruritus. | ^156,157^ |
| NCT03757858 | The objective response rate (RR) was 10 of 33, including 3 with a CR and 7 with a PR. The disease control rate (DCR) was 22 of 33. The DCR was 7 of 10, 6 of 11, and 9 of 12 in HT+ACT, HT+ACT+PD-1 and  HT+ACT+CT groups respectively. | IL-2, IL-4, TNF-a, and IFN-c levels in peripheral blood were significantly increased among the clinical responders while IL-6 and IL-10 were elevated among those with progressive disease.  Peripheral blood CD8+/CD28+ T cells increased, while the CD4+/CD25+/CD127+Treg cells  decreased after therapy. TCR diversity was substantially increased among the clinical responders. | The most common adverse reactions, blistering, subcutaneous fat induration, local heat-related pain, vomiting and sinus tachycardia, were observed in association with HT. No immune-mediated adverse events | ^158^ |
| NCT03080974 | Mean time to progression was 6.3  months with current median overall survival of 18.0 months. | T-effector memory cells increased from baseline by 1.96-fold by postoperative day 90. No differences  were noted among absolute cell counts of CD4+ T cells, naïve T cells,  or T-central memory cells. | No dose-limiting toxicities occurred.  Seven patients developed grade 3/4 treatment-related adverse events;  nivolumab-related adverse events occurred in 1 patient. | ^159^ |

# Supplemental Table S6. Results of FT+ICI clinical studies for cancers

**Reference:**

1 den Brok, M. *et al.* In situ tumor ablation creates an antigen source for the generation of antitumor immunity. *CANCER RESEARCH* **64**, 4024-4029, doi:10.1158/0008-5472.CAN-03-3949 (2004).

2 den Brok, M. *et al.* Synergy between in situ cryoablation and TLR9 stimulation results in a highly effective in vivo dendritic cell vaccine. *CANCER RESEARCH* **66**, 7285-7292, doi:10.1158/0008-5472.CAN-06-0206 (2006).

3 Shi, L. R. *et al.* PD-1 Blockade Boosts Radiofrequency Ablation-Elicited Adaptive Immune Responses against Tumor. *CLINICAL CANCER RESEARCH* **22**, 1173-1184, doi:10.1158/1078-0432.CCR-15-1352 (2016).

4 Shi, L. R. *et al.* Inflammation induced by incomplete radiofrequency ablation accelerates tumor progression and hinders PD-1 immunotherapy. *NATURE COMMUNICATIONS* **10**, doi:10.1038/s41467-019-13204-3 (2019).

5 Han, X. *et al.* In situ thermal ablation of tumors in combination with nano-adjuvant and immune checkpoint blockade to inhibit cancer metastasis and recurrence. *Biomaterials* **224**, 119490, doi:10.1016/j.biomaterials.2019.119490 (2019).

6 Zhang, L. *et al.* Antitumor Immunity Augmented by Combining Radiofrequency Ablation with Anti-CTLA-4 Therapy in a Subcutaneous Murine Hepatoma Model. *JOURNAL OF VASCULAR AND INTERVENTIONAL RADIOLOGY* **31**, 1178-1186, doi:10.1016/j.jvir.2020.01.022 (2020).

7 Zhang, L., Wang, J., Jiang, J. H., Zhang, M. M. & Shen, J. L. CTLA-4 Blockade Suppresses Progression of Residual Tumors and Improves Survival After Insufficient Radiofrequency Ablation in a Subcutaneous Murine Hepatoma Model. *CARDIOVASCULAR AND INTERVENTIONAL RADIOLOGY* **43**, 1353-1361, doi:10.1007/s00270-020-02505-6 (2020).

8 Chen, Z. B., Shen, S. Q., Peng, B. G. & Tao, J. P. Intratumoural GM-CSF microspheres and CTLA-4 blockade enhance the antitumour immunity induced by thermal ablation in a subcutaneous murine hepatoma model. *INTERNATIONAL JOURNAL OF HYPERTHERMIA* **25**, 374-382, doi:10.1080/02656730902976807 (2009).

9 Zhu, J. *et al.* Enhanced antitumor efficacy through microwave ablation in combination with immune checkpoints blockade in breast cancer: A pre-clinical study in a murine model. *DIAGNOSTIC AND INTERVENTIONAL IMAGING* **99**, 135-142, doi:10.1016/j.diii.2017.12.011 (2018).

10 Duan, X. *et al.* Combined use of microwave ablation and cell immunotherapy induces nonspecific immunity of hepatocellular carcinoma model mice. *Cell Cycle* **19**, 3595-3607, doi:10.1080/15384101.2020.1853942 (2020).

11 Huang, S. J. *et al.* Microwave ablation combined with anti-PD-1 therapy enhances systemic antitumor immunity in a multitumor murine model of Hepa1-6. *INTERNATIONAL JOURNAL OF HYPERTHERMIA* **39**, 278-286, doi:10.1080/02656736.2022.2032406 (2022).

12 Shao, D. *et al.* LAG3 blockade coordinates with microwave ablation to promote CD8(+) T cell-mediated anti-tumor immunity. *JOURNAL OF TRANSLATIONAL MEDICINE* **20**, doi:10.1186/s12967-022-03646-7 (2022).

13 Silvestrini, M. T. *et al.* Priming is key to effective incorporation of image-guided thermal ablation into immunotherapy protocols. *JCI Insight* **2**, e90521, doi:10.1172/jci.insight.90521 (2017).

14 Chavez, M. *et al.* Distinct immune signatures in directly treated and distant tumors result from TLR adjuvants and focal ablation. *THERANOSTICS* **8**, 3611-3628, doi:10.7150/thno.25613 (2018).

15 Sheybani, N. D. *et al.* Combination of thermally ablative focused ultrasound with gemcitabine controls breast cancer via adaptive immunity. *JOURNAL FOR IMMUNOTHERAPY OF CANCER* **8**, doi:10.1136/jitc-2020-001008 (2020).

16 Fite, B. Z. *et al.* Immune modulation resulting from MR-guided high intensity focused ultrasound in a model of murine breast cancer. *SCIENTIFIC REPORTS* **11**, doi:10.1038/s41598-020-80135-1 (2021).

17 Mouratidis, P. X. E., Costa, M., Rivens, I., Repasky, E. E. & ter Haar, G. Pulsed focused ultrasound can improve the anti-cancer effects of immune checkpoint inhibitors in murine pancreatic cancer. *JOURNAL OF THE ROYAL SOCIETY INTERFACE* **18**, doi:10.1098/rsif.2021.0266 (2021).

18 den Brok, M. H. *et al.* Efficient loading of dendritic cells following cryo and radiofrequency ablation in combination with immune modulation induces anti-tumour immunity. *British journal of cancer* **95**, 896-905, doi:10.1038/sj.bjc.6603341 (2006).

19 Waitz, R. *et al.* Potent Induction of Tumor Immunity by Combining Tumor Cryoablation with Anti-CTLA-4 Therapy. *CANCER RESEARCH* **72**, 430-439, doi:10.1158/0008-5472.CAN-11-1782 (2012).

20 Li, F. *et al.* Anti-tumor immunological response induced by cryoablation and anti-CTLA-4 antibody in an in vivo RM-1 cell prostate cancer murine model. *Neoplasma* **61**, 659-671, doi:10.4149/neo_2014_081 (2014).

21 Kudo-Saito, C., Fuwa, T. & Kawakami, Y. Targeting ALCAM in the cryo-treated tumour microenvironment successfully induces systemic anti-tumour immunity. *EUROPEAN JOURNAL OF CANCER* **62**, 54-61, doi:10.1016/j.ejca.2016.04.013 (2016).

22 Benzon, B. *et al.* Combining immune check-point blockade and cryoablation in an immunocompetent hormone sensitive murine model of prostate cancer. *PROSTATE CANCER AND PROSTATIC DISEASES* **21**, 126-136, doi:10.1038/s41391-018-0035-z (2018).

23 Zhu, C., Lin, S., Liang, J. & Zhu, Y. PD-1 blockade enhances the anti-tumor immune response induced by cryoablation in a murine model of renal cell carcinoma. *Cryobiology* **87**, 86-90, doi:10.1016/j.cryobiol.2019.01.015 (2019).

24 Annen, R. *et al.* Tumor-Specific Immunoenhancing Effects after Local Cryoablation for Metastatic Bone Tumor in a Mouse Model. *INTERNATIONAL JOURNAL OF MOLECULAR SCIENCES* **23**, doi:10.3390/ijms23169445 (2022).

25 Tan, J. *et al.* Anti-PD-L1 antibody enhances curative effect of cryoablation via antibody-dependent cell-mediated cytotoxicity mediating PD-L1highCD11b+ cells elimination in hepatocellular carcinoma. *Acta Pharmaceutica Sinica B*, doi:<https://doi.org/10.1016/j.apsb.2022.08.006> (2022).

26 Yu, Z.-P., Sun, X.-W., He, Y.-P., Gu, J. & Jin, Y. PD-1 monoclonal antibodies enhance the cryoablation-induced antitumor immune response: a breast cancer murine model research. *International Journal of Hyperthermia* **40**, 2164625, doi:10.1080/02656736.2022.2164625 (2023).

27 Narayanan, J. S. S. *et al.* Irreversible Electroporation Combined with Checkpoint Blockade and TLR7 Stimulation Induces Antitumor Immunity in a Murine Pancreatic Cancer Model. *Cancer Immunology Research* **7**, 1714-1726, doi:10.1158/2326-6066.CIR-19-0101 (2019).

28 Zhao, J. *et al.* Irreversible electroporation reverses resistance to immune checkpoint blockade in pancreatic cancer. *Nature Communications* **10**, 899, doi:10.1038/s41467-019-08782-1 (2019).

29 Qian, J. J. *et al.* Blocking exposed PD-L1 elicited by nanosecond pulsed electric field reverses dysfunction of CD8(+) T cells in liver cancer. *CANCER LETTERS* **495**, 1-11, doi:10.1016/j.canlet.2020.09.015 (2020).

30 Burbach, B. J. *et al.* Irreversible electroporation augments checkpoint immunotherapy in prostate cancer and promotes tumor antigen-specific tissue-resident memory CD8+ T cells. *Nature Communications* **12**, 3862, doi:10.1038/s41467-021-24132-6 (2021).

31 Babikr, F. *et al.* Distinct roles but cooperative effect of TLR3/9 agonists and PD-1 blockade in converting the immunotolerant microenvironment of irreversible electroporation-ablated tumors. *CELLULAR & MOLECULAR IMMUNOLOGY* **18**, 2632-2647, doi:10.1038/s41423-021-00796-4 (2021).

32 Shi, X. J. *et al.* Irreversible electroporation enhances immunotherapeutic effect in the off-target tumor in a murine model of orthotopic HCC. *AMERICAN JOURNAL OF CANCER RESEARCH* **11**, 3304-3319 (2021).

33 Bulner, S., Prodeus, A., Gariepy, J., Hynynen, K. & Goertz, D. E. ENHANCING CHECKPOINT INHIBITOR THERAPY WITH ULTRASOUND STIMULATED MICROBUBBLES. *Ultrasound in Medicine and Biology* **45**, 500-512, doi:10.1016/j.ultrasmedbio.2018.10.002 (2019).

34 Eranki, A. *et al.* High-Intensity Focused Ultrasound (HIFU) Triggers Immune Sensitization of Refractory Murine Neuroblastoma to Checkpoint Inhibitor Therapy. *CLINICAL CANCER RESEARCH* **26**, 1152-1161, doi:10.1158/1078-0432.CCR-19-1604 (2020).

35 Nam, G. H. *et al.* Investigation of the Potential Immunological Effects of Boiling Histotripsy for Cancer Treatment. *ADVANCED THERAPEUTICS* **3**, doi:10.1002/adtp.201900214 (2020).

36 Qu, S. B. *et al.* Non-thermal histotripsy tumor ablation promotes abscopal immune responses that enhance cancer immunotherapy. *JOURNAL FOR IMMUNOTHERAPY OF CANCER* **8**, doi:10.1136/jitc-2019-000200 (2020).

37 Abe, S. *et al.* Combination of ultrasound-based mechanical disruption of tumor with immune checkpoint blockade modifies tumor microenvironment and augments systemic antitumor immunity. *JOURNAL FOR IMMUNOTHERAPY OF CANCER* **10**, doi:10.1136/jitc-2021-003717 (2022).

38 Hayashi, F. *et al.* Combined Treatment with Ultrasound and Immune Checkpoint Inhibitors for Prostate Cancer. *JOURNAL OF CLINICAL MEDICINE* **11**, doi:10.3390/jcm11092448 (2022).

39 Ibuki, Y. *et al.* Local hyperthermia combined with CTLA-4 blockade induces both local and abscopal effects in a murine breast cancer model. *INTERNATIONAL JOURNAL OF HYPERTHERMIA* **38**, 363-371, doi:10.1080/02656736.2021.1875059 (2021).

40 Wang, C. *et al.* Immunological Responses Triggered by Photothermal Therapy with Carbon Nanotubes in Combination with Anti-CTLA-4 Therapy to Inhibit Cancer Metastasis. *ADVANCED MATERIALS* **26**, 8154-8162, doi:10.1002/adma.201402996 (2014).

41 Chen, Q. *et al.* Photothermal therapy with immune-adjuvant nanoparticles together with checkpoint blockade for effective cancer immunotherapy. *NATURE COMMUNICATIONS* **7**, doi:10.1038/ncomms13193 (2016).

42 Cano-Mejia, J. *et al.* Prussian blue nanoparticle-based photothermal therapy combined with checkpoint inhibition for photothermal immunotherapy of neuroblastoma. *NANOMEDICINE-NANOTECHNOLOGY BIOLOGY AND MEDICINE* **13**, 771-781, doi:10.1016/j.nano.2016.10.015 (2017).

43 Liu, Y. *et al.* Synergistic Immuno Photothermal Nanotherapy (SYMPHONY) for the Treatment of Unresectable and Metastatic Cancers. *SCIENTIFIC REPORTS* **7**, doi:10.1038/s41598-017-09116-1 (2017).

44 Ge, R. *et al.* Photothermal-Activatable Fe3O4 Superparticle Nanodrug Carriers with PD-L1 Immune Checkpoint Blockade for Anti-metastatic Cancer Immunotherapy. *ACS APPLIED MATERIALS & INTERFACES* **10**, 20342-20355, doi:10.1021/acsami.8b05876 (2018).

45 Luo, L. H. *et al.* Laser Immunotherapy in Combination with Perdurable PD-1 Blocking for the Treatment of Metastatic Tumors. *ACS NANO* **12**, 7647-7662, doi:10.1021/acsnano.8b00204 (2018).

46 Zhou, B. Q. *et al.* in *BIOPHOTONICS AND IMMUNE RESPONSES XIV* Vol. 10879 (2019).

47 Wang, T. T. *et al.* A cancer vaccine-mediated postoperative immunotherapy for recurrent and metastatic tumors. *NATURE COMMUNICATIONS* **9**, doi:10.1038/s41467-018-03915-4 (2018).

48 Chang, M. Y. *et al.* A Multifunctional Cascade Bioreactor Based on Hollow-Structured Cu2MoS4 for Synergetic Cancer Chemo-Dynamic Therapy/Starvation Therapy/Phototherapy/Immunotherapy with Remarkably Enhanced Efficacy. *ADVANCED MATERIALS* **31**, doi:10.1002/adma.201905271 (2019).

49 Chen, L. *et al.* Tumor-Targeted Drug and CpG Delivery System for Phototherapy and Docetaxel-Enhanced Immunotherapy with Polarization toward M1-Type Macrophages on Triple Negative Breast Cancers. *ADVANCED MATERIALS* **31**, doi:10.1002/adma.201904997 (2019).

50 Chen, W. *et al.* Combination of Bacterial‐Photothermal Therapy with an Anti‐PD‐1 Peptide Depot for Enhanced Immunity against Advanced Cancer. *Advanced Functional Materials* **30**, doi:10.1002/adfm.201906623 (2019).

51 Han, X. *et al.* Platelets as platforms for inhibition of tumor recurrence post-physical therapy by delivery of anti-PD-L1 checkpoint antibody. *JOURNAL OF CONTROLLED RELEASE* **304**, 233-241, doi:10.1016/j.jconrel.2019.05.008 (2019).

52 He, L. *et al.* Designing Bioinspired 2D MoSe2 Nanosheet for Efficient Photothermal‐Triggered Cancer Immunotherapy with Reprogramming Tumor‐Associated Macrophages. *Advanced Functional Materials* **29**, doi:10.1002/adfm.201901240 (2019).

53 Li, Y. *et al.* Phototherapy using immunologically modified carbon nanotubes to potentiate checkpoint blockade for metastatic breast cancer. *Nanomedicine-Nanotechnology Biology and Medicine* **18**, 44-53, doi:10.1016/j.nano.2019.02.009 (2019).

54 Liang, X. *et al.* Photothermal cancer immunotherapy by erythrocyte membrane-coated black phosphorus formulation. *JOURNAL OF CONTROLLED RELEASE* **296**, 150-161, doi:10.1016/j.jconrel.2019.01.027 (2019).

55 Liu, B. *et al.* Effects of gold nanoprism-assisted human PD-L1 siRNA on both gene down-regulation and photothermal therapy on lung cancer. *ACTA BIOMATERIALIA* **99**, 307-319, doi:10.1016/j.actbio.2019.08.046 (2019).

56 Lu, Q. L. *et al.* Photothermally activatable PDA immune nanomedicine combined with PD-L1 checkpoint blockade for antimetastatic cancer photoimmunotherapy. *JOURNAL OF MATERIALS CHEMISTRY B* **7**, 2499-2511, doi:10.1039/c9tb00089e (2019).

57 Peng, J. *et al.* Tumor Microenvironment Responsive Drug‐Dye‐Peptide Nanoassembly for Enhanced Tumor‐Targeting, Penetration, and Photo‐Chemo‐Immunotherapy. *Advanced Functional Materials* **29**, doi:10.1002/adfm.201900004 (2019).

58 Song, Y. L. *et al.* Immune-adjuvant loaded Bi2Se3 nanocage for photothermal-improved PD-L1 checkpoint blockade immune-tumor metastasis therapy. *Nano Research* **12**, 1770-1780, doi:10.1007/s12274-019-2341-8 (2019).

59 Sun, W. *et al.* Synergistic triple-combination therapy with hyaluronic acid-shelled PPy/CPT nanoparticles results in tumor regression and prevents tumor recurrence and metastasis in 4T1 breast cancer. *Biomaterials* **217**, 119264, doi:10.1016/j.biomaterials.2019.119264 (2019).

60 Tian, Y. *et al.* JQ1-Loaded Polydopamine Nanoplatform Inhibits c-MYC/Programmed Cell Death Ligand 1 to Enhance Photothermal Therapy for Triple-Negative Breast Cancer. *ACS APPLIED MATERIALS & INTERFACES* **11**, 46626-46636, doi:10.1021/acsami.9b18730 (2019).

61 Wang, R. P. *et al.* Surface-Functionalized Modified Copper Sulfide Nanoparticles Enhance Checkpoint Blockade Tumor Immunotherapy by Photothermal Therapy and Antigen Capturing. *ACS APPLIED MATERIALS & INTERFACES* **11**, 13964-13972, doi:10.1021/acsami.9b01107 (2019).

62 Wen, M. *et al.* Artificial Enzyme Catalyzed Cascade Reactions: Antitumor Immunotherapy Reinforced by NIR-II Light. *ANGEWANDTE CHEMIE-INTERNATIONAL EDITION* **58**, 17425-17432, doi:10.1002/anie.201909729 (2019).

63 Wen, Y. Y. *et al.* Photothermal-Chemotherapy Integrated Nanoparticles with Tumor Microenvironment Response Enhanced the Induction of Immunogenic Cell Death for Colorectal Cancer Efficient Treatment. *ACS APPLIED MATERIALS & INTERFACES* **11**, 43393-43408, doi:10.1021/acsami.9b17137 (2019).

64 Yan, M. M. *et al.* Nanoscale Reduced Graphene Oxide-Mediated Photothermal Therapy Together with IDO Inhibition and PD-L1 Blockade Synergistically Promote Antitumor Immunity. *ACS APPLIED MATERIALS & INTERFACES* **11**, 1876-1885, doi:10.1021/acsami.8b18751 (2019).

65 Yan, S. Q. *et al.* Activating Antitumor Immunity and Antimetastatic Effect Through Polydopamine-Encapsulated Core-Shell Upconversion Nanoparticles. *ADVANCED MATERIALS* **31**, doi:10.1002/adma.201905825 (2019).

66 Yang, Q. *et al.* Rationally designed peptide-conjugated gold/platinum nanosystem with active tumor-targeting for enhancing tumor photothermal-immunotherapy. *JOURNAL OF CONTROLLED RELEASE* **308**, 29-43, doi:10.1016/j.jconrel.2019.06.031 (2019).

67 Ye, X. Y. *et al.* Surgical Tumor-Derived Personalized Photothermal Vaccine Formulation for Cancer Immunotherapy. *ACS NANO* **13**, 2956-2968, doi:10.1021/acsnano.8b07371 (2019).

68 Zhang, D. *et al.* Tumor microenvironment responsive FePt/MoS2 nanocomposites with chemotherapy and photothermal therapy for enhancing cancer immunotherapy. *Nanoscale* **11**, 19912-19922, doi:10.1039/c9nr05684j (2019).

69 Zhang, N. *et al.* Photothermal therapy mediated by phase-transformation nanoparticles facilitates delivery of anti-PD1 antibody and synergizes with antitumor immunotherapy for melanoma. *JOURNAL OF CONTROLLED RELEASE* **306**, 15-28, doi:10.1016/j.jconrel.2019.05.036 (2019).

70 Cano-Mejia, J. *et al.* CpG-coated prussian blue nanoparticles-based photothermal therapy combined with anti-CTLA-4 immune checkpoint blockade triggers a robust abscopal effect against neuroblastoma. *TRANSLATIONAL ONCOLOGY* **13**, doi:10.1016/j.tranon.2020.100823 (2020).

71 Cao, Q. *et al.* Induction of antitumor immunity in mice by the combination of nanoparticle-based photothermolysis and anti-PD-1 checkpoint inhibition. *Nanomedicine* **25**, 102169, doi:10.1016/j.nano.2020.102169 (2020).

72 Chen, H. W. *et al.* Depleting tumor-associated Tregs via nanoparticle-mediated hyperthermia to enhance anti-CTLA-4 immunotherapy. *NANOMEDICINE* **15**, 77-92, doi:10.2217/nnm-2019-0190 (2020).

73 Dong, X. *et al.* A Heterojunction Structured WO2.9-WSe2 Nanoradiosensitizer Increases Local Tumor Ablation and Checkpoint Blockade Immunotherapy upon Low Radiation Dose. *ACS Nano* **14**, 5400-5416, doi:10.1021/acsnano.9b08962 (2020).

74 He, C., Yu, L., Yao, H., Chen, Y. & Hao, Y. Combinatorial Photothermal 3D‐Printing Scaffold and Checkpoint Blockade Inhibits Growth/Metastasis of Breast Cancer to Bone and Accelerates Osteogenesis. *Advanced Functional Materials* **31**, doi:10.1002/adfm.202006214 (2020).

75 Hu, C. *et al.* Copper-Doped Nanoscale Covalent Organic Polymer for Augmented Photo/Chemodynamic Synergistic Therapy and Immunotherapy. *Bioconjugate Chemistry* **31**, 1661-1670, doi:10.1021/acs.bioconjchem.0c00209 (2020).

76 Huang, T. Y. *et al.* Supramolecular Photothermal Nanomedicine Mediated Distant Tumor Inhibition via PD-1 and TIM-3 Blockage. *FRONTIERS IN CHEMISTRY* **8**, doi:10.3389/fchem.2020.00001 (2020).

77 Li, X. Y. *et al.* Biogenic Hybrid Nanosheets Activated Photothermal Therapy and Promoted Anti-PD-L1 Efficacy for Synergetic Antitumor Strategy. *ACS APPLIED MATERIALS & INTERFACES* **12**, 29122-29132, doi:10.1021/acsami.0c09111 (2020).

78 Liu, Y. *et al.* Tumor microenvironment-responsive prodrug nanoplatform via co-self-assembly of photothermal agent and IDO inhibitor for enhanced tumor penetration and cancer immunotherapy. *Biomaterials* **242**, 119933, doi:10.1016/j.biomaterials.2020.119933 (2020).

79 Zeng, F. C. *et al.* Photoacoustic-immune therapy with a multi-purpose black phosphorus-based nanoparticle. *NANO RESEARCH* **13**, 3403-3415, doi:10.1007/s12274-020-3028-x (2020).

80 Zhang, M. *et al.* Biodegradable Poly(gamma-glutamic acid)@glucose oxidase@carbon dot nanoparticles for simultaneous multimodal imaging and synergetic cancer therapy. *Biomaterials* **252**, 120106, doi:10.1016/j.biomaterials.2020.120106 (2020).

81 Zhang, Y. *et al.* Plasmonic modulation of gold nanotheranostics for targeted NIR-II photothermal-augmented immunotherapy. *Nano Today* **35**, doi:10.1016/j.nantod.2020.100987 (2020).

82 Zhao, J. *et al.* Effect of microwave ablation treatment of hepatic malignancies on serum cytokine levels. *BMC Cancer* **20**, 812, doi:10.1186/s12885-020-07326-x (2020).

83 Zhou, Y., Liu, S. N., Hu, C. L., Cai, L. H. & Pang, M. L. A covalent organic framework as a nanocarrier for synergistic phototherapy and immunotherapy. *JOURNAL OF MATERIALS CHEMISTRY B* **8**, 5451-5459, doi:10.1039/d0tb00679c (2020).

84 Fang, X. Y. *et al.* Biomimetic Anti-PD-1 Peptide-Loaded 2D FePSe3 Nanosheets for Efficient Photothermal and Enhanced Immune Therapy with Multimodal MR/PA/Thermal Imaging. *ADVANCED SCIENCE* **8**, doi:10.1002/advs.202003041 (2021).

85 Feng, Z. H. *et al.* A combination strategy based on an Au nanorod/doxorubicin gel via mild photothermal therapy combined with antigen-capturing liposomes and anti-PD-L1 agent promote a positive shift in the cancer-immunity cycle. *ACTA BIOMATERIALIA* **136**, 495-507, doi:10.1016/j.actbio.2021.09.052 (2021).

86 Gao, T. *et al.* Reshaping Antitumor Immunity with Chemo-Photothermal Integrated Nanoplatform to Augment Checkpoint Blockade-Based Cancer Therapy. *ADVANCED FUNCTIONAL MATERIALS* **31**, doi:10.1002/adfm.202100437 (2021).

87 He, C., Yu, L. D., Yao, H. L., Chen, Y. & Hao, Y. Q. Combinatorial Photothermal 3D-Printing Scaffold and Checkpoint Blockade Inhibits Growth/Metastasis of Breast Cancer to Bone and Accelerates Osteogenesis. *ADVANCED FUNCTIONAL MATERIALS* **31**, doi:10.1002/adfm.202006214 (2021).

88 Huang, X. Y. *et al.* Combined photothermal-immunotherapy via poly-tannic acid coated PLGA nanoparticles for cancer treatment. *BIOMATERIALS SCIENCE* **9**, 6282-6294, doi:10.1039/d1bm00474c (2021).

89 Jin, L. J., Shen, S., Huang, Y. J., Li, D. D. & Yang, X. Z. Corn-like Au/Ag nanorod-mediated NIR-II photothermal/photodynamic therapy potentiates immune checkpoint antibody efficacy by reprogramming the cold tumor microenvironment. *BIOMATERIALS* **268**, doi:10.1016/j.biomaterials.2020.120582 (2021).

90 Li, W. H. *et al.* Black phosphorous nanosheet: A novel immune-potentiating nanoadjuvant for near-infrared-improved immunotherapy. *Biomaterials* **273**, 120788, doi:10.1016/j.biomaterials.2021.120788 (2021).

91 Liu, S. S. *et al.* Fe2P nanorods based photothermal therapy combined with immune checkpoint inhibitors for pancreatic cancer. *NANOPHOTONICS* **10**, 3267-3278, doi:10.1515/nanoph-2021-0196 (2021).

92 Liu, X. W. *et al.* Combination of MAPK inhibition with photothermal therapy synergistically augments the anti-tumor efficacy of immune checkpoint blockade. *JOURNAL OF CONTROLLED RELEASE* **332**, 194-209, doi:10.1016/j.jconrel.2021.02.020 (2021).

93 Liu, Y. *et al.* Plasmonic gold nanostars for synergistic photoimmunotherapy to treat cancer. *Nanophotonics* **10**, 3295-3302, doi:10.1515/nanoph-2021-0237 (2021).

94 McKernan, P. *et al.* Targeted Single-Walled Carbon Nanotubes for Photothermal Therapy Combined with Immune Checkpoint Inhibition for the Treatment of Metastatic Breast Cancer. *NANOSCALE RESEARCH LETTERS* **16**, doi:10.1186/s11671-020-03459-x (2021).

95 Noh, I. *et al.* Targeting the tumor microenvironment with amphiphilic near-infrared cyanine nanoparticles for potentiated photothermal immunotherapy. *Biomaterials* **275**, 120926, doi:10.1016/j.biomaterials.2021.120926 (2021).

96 Tan, Y. N. *et al.* Thermal-sensitive lipid nanoparticles potentiate anti-PD therapy through enhancing drug penetration and T lymphocytes infiltration in metastatic tumor. *CANCER LETTERS* **522**, 238-254, doi:10.1016/j.canlet.2021.09.031 (2021).

97 Wang, Q., Niu, D. G., Shi, J. S. & Wang, L. L. A Three-in-one ZIFs-Derived CuCo(O)/GOx@PCNs Hybrid Cascade Nanozyme for Immunotherapy/Enhanced Starvation/Photothermal Therapy. *ACS APPLIED MATERIALS & INTERFACES* **13**, 11683-11695, doi:10.1021/acsami.1c01006 (2021).

98 Wang, S. Y. *et al.* Photothermal therapy mediated by gold nanocages composed of anti-PDL1 and galunisertib for improved synergistic immunotherapy in colorectal cancer. *ACTA BIOMATERIALIA* **134**, 621-632, doi:10.1016/j.actbio.2021.07.051 (2021).

99 Yang, P. *et al.* Effective Photothermal Therapy Mediated by Indocyanine Green Nanoparticle Tip-Loaded Microneedles to Enhance Checkpoint Inhibitor Immunotherapy for Melanoma Treatment. *ACS Applied Nano Materials* **4**, 5921-5931, doi:10.1021/acsanm.1c00832 (2021).

100 Yu, J. *et al.* Combining PD-L1 inhibitors with immunogenic cell death triggered by chemo-photothermal therapy via a thermosensitive liposome system to stimulate tumor-specific immunological response. *NANOSCALE* **13**, 12966-12978, doi:10.1039/d1nr03288g (2021).

101 Yu, Q. W. *et al.* Mild hyperthermia promotes immune checkpoint blockade-based immunotherapy against metastatic pancreatic cancer using size-adjustable nanoparticles. *ACTA BIOMATERIALIA* **133**, 244-256, doi:10.1016/j.actbio.2021.05.002 (2021).

102 Zhao, P. Q. *et al.* Biomimetic black phosphorus quantum dots-based photothermal therapy combined with anti-PD-L1 treatment inhibits recurrence and metastasis in triple-negative breast cancer. *JOURNAL OF NANOBIOTECHNOLOGY* **19**, doi:10.1186/s12951-021-00932-2 (2021).

103 Zhao, W. R. *et al.* M2-Like TAMs Function Reversal Contributes to Breast Cancer Eradication by Combination Dual Immune Checkpoint Blockade and Photothermal Therapy. *SMALL* **17**, doi:10.1002/smll.202007051 (2021).

104 Zhao, X. *et al.* Combining photothermal ablation-based vaccine with immune checkpoint blockade for synergistic osteosarcoma immunotherapy. *Materials & Design* **198**, doi:10.1016/j.matdes.2020.109311 (2021).

105 Zhou, M. *et al.* Combining Photothermal Therapy-Induced Immunogenic Cell Death and Hypoxia Relief-Benefited M1-Phenotype Macrophage Polarization for Cancer Immunotherapy. *ADVANCED THERAPEUTICS* **4**, doi:10.1002/adtp.202000191 (2021).

106 Cao, Y. B. *et al.* Nanoplatform Self-Assembly from Small Molecules of Porphyrin Derivatives for NIR-II Fluorescence Imaging Guided Photothermal-Immunotherapy. *ADVANCED HEALTHCARE MATERIALS* **11**, doi:10.1002/adhm.202102526 (2022).

107 Chen, Y. *et al.* Glutathione-Depleting Organic Metal Adjuvants for Effective NIR-II Photothermal Immunotherapy. *ADVANCED MATERIALS* **34**, doi:10.1002/adma.202201706 (2022).

108 Du, Y. *et al.* NIR-II fluorescence imaging-guided hepatocellular carcinoma treatment via IR-1061-acridine and lenvatinib co-loaded thermal-sensitive micelles and anti-PD-1 combinational therapy. *CHEMICAL ENGINEERING JOURNAL* **454**, doi:10.1016/j.cej.2022.140437 (2023).

109 Gao, Y. *et al.* Engineering Platelets with PDL1 Antibodies and Iron Oxide Nanoparticles for Postsurgical Cancer Immunotherapy. *ACS APPLIED BIO MATERIALS*, doi:10.1021/acsabm.2c00869 (2022).

110 Jin, C. Y., Zhang, Y., Zhang, G. X., Wang, B. & Hua, P. Y. Combination of GNRs-PEI/cGAMP-laden macrophages-based photothermal induced in situ tumor vaccines and immune checkpoint blockade for synergistic anti-tumor immunotherapy. *BIOMATERIALS ADVANCES* **133**, doi:10.1016/j.msec.2021.112603 (2022).

111 Lin, X. H. *et al.* Biomimetic nanoprobe-augmented triple therapy with photothermal, sonodynamic and checkpoint blockade inhibits tumor growth and metastasis. *JOURNAL OF NANOBIOTECHNOLOGY* **20**, doi:10.1186/s12951-022-01287-y (2022).

112 Liu, H. S. *et al.* Metal-organic framework-mediated multifunctional nanoparticles for combined chemo-photothermal therapy and enhanced immunotherapy against colorectal cancer. *ACTA BIOMATERIALIA* **144**, 132-141, doi:10.1016/j.actbio.2022.03.023 (2022).

113 Lu, Y. F. *et al.* Ultra-thin layered double hydroxide-mediated photothermal therapy combine with asynchronous blockade of PD-L1 and NR2F6 inhibit hepatocellular carcinoma. *JOURNAL OF NANOBIOTECHNOLOGY* **20**, doi:10.1186/s12951-022-01565-9 (2022).

114 Ning, B. *et al.* Low-temperature photothermal irradiation triggers alkyl radicals burst for potentiating cancer immunotherapy. *JOURNAL OF COLLOID AND INTERFACE SCIENCE* **614**, 436-450, doi:10.1016/j.jcis.2022.01.131 (2022).

115 Pu, Y. Y. *et al.* Starvation therapy enabled "switch-on" NIR-II photothermal nanoagent for synergistic in situ photothermal immunotherapy. *NANO TODAY* **44**, doi:10.1016/j.nantod.2022.101461 (2022).

116 Xiang, Q. Q. *et al.* Near-Infrared II Nanoadjuvant-Mediated Chemodynamic, Photodynamic, and Photothermal Therapy Combines Immunogenic Cell Death with PD-L1 Blockade to Enhance Antitumor Immunity. *SMALL* **18**, doi:10.1002/smll.202107809 (2022).

117 Yan, D. Y. *et al.* Multimodal Imaging-Guided Photothermal Immunotherapy Based on a Versatile NIR-II Aggregation-Induced Emission Luminogen. *ANGEWANDTE CHEMIE-INTERNATIONAL EDITION* **61**, doi:10.1002/anie.202202614 (2022).

118 Yasothamani, V. & Vivek, R. Targeted NIR-responsive theranostic immuno-nanomedicine combined TLR7 agonist with immune checkpoint blockade for effective cancer photothermal immunotherapy. *JOURNAL OF MATERIALS CHEMISTRY B* **10**, 6392-6403, doi:10.1039/d2tb01195f (2022).

119 Yu, Y. J. *et al.* Polymeric PD-L1 blockade nanoparticles for cancer photothermal-immunotherapy. *BIOMATERIALS* **280**, doi:10.1016/j.biomaterials.2021.121312 (2022).

120 Zhang, Y. *et al.* Mitochondrial targeted melanin@mSiO(2) yolk-shell nanostructures for NIR-II-driven photo-thermal-dynamic/immunotherapy. *CHEMICAL ENGINEERING JOURNAL* **435**, doi:10.1016/j.cej.2022.134869 (2022).

121 Oei, A. L. *et al.* Enhancing the abscopal effect of radiation and immune checkpoint inhibitor therapies with magnetic nanoparticle hyperthermia in a model of metastatic breast cancer. *INTERNATIONAL JOURNAL OF HYPERTHERMIA* **36**, 47-63, doi:10.1080/02656736.2019.1685686 (2019).

122 Chao, Y. *et al.* Iron Nanoparticles for Low-Power Local Magnetic Hyperthermia in Combination with Immune Checkpoint Blockade for Systemic Antitumor Therapy. *Nano Letters* **19**, 4287-4296, doi:10.1021/acs.nanolett.9b00579 (2019).

123 Liu, X. *et al.* Ferrimagnetic Vortex Nanoring-Mediated Mild Magnetic Hyperthermia Imparts Potent Immunological Effect for Treating Cancer Metastasis. *ACS Nano* **13**, 8811-8825, doi:10.1021/acsnano.9b01979 (2019).

124 Wang, Z. *et al.* Janus Nanobullets Combine Photodynamic Therapy and Magnetic Hyperthermia to Potentiate Synergetic Anti-Metastatic Immunotherapy. *ADVANCED SCIENCE* **6**, doi:10.1002/advs.201901690 (2019).

125 Pan, J. *et al.* Combined Magnetic Hyperthermia and Immune Therapy for Primary and Metastatic Tumor Treatments. *ACS Nano* **14**, 1033-1044, doi:10.1021/acsnano.9b08550 (2020).

126 Chalmers, Z. R. *et al.* Analysis of 100,000 human cancer genomes reveals the landscape of tumor mutational burden. *GENOME MEDICINE* **9**, doi:10.1186/s13073-017-0424-2 (2017).

127 braintumor.org. *About Glioblastoma*, <<https://braintumor.org/events/glioblastoma-awareness-day/about-glioblastoma/>> (

128 cancer.org. *Survival Rates for Pancreatic Cancer*, <<https://www.cancer.org/cancer/pancreatic-cancer/detection-diagnosis-staging/survival-rates.html>> (

129 cancer.org. *Survival Rates for Bile Duct Cancer*, <<https://www.cancer.org/cancer/bile-duct-cancer/detection-diagnosis-staging/survival-by-stage.html>> (

130 cancer.net. *Lung Cancer - Non-Small Cell: Statistics*, <<https://www.cancer.net/cancer-types/lung-cancer-non-small-cell/statistics>> (

131 cancer.org. *Lung Cancer Survival Rates*, <<https://www.cancer.org/cancer/bile-duct-cancer/detection-diagnosis-staging/survival-by-stage.html>> (

132 cancer.net. *Colorectal Cancer: Statistics*, <<https://www.cancer.net/cancer-types/colorectal-cancer/statistics>> (

133 cancer.org. *Survival Rates for Kidney Cancer*, <<https://www.cancer.org/cancer/kidney-cancer/detection-diagnosis-staging/survival-rates.html>> (

134 cancer.net. *Lymphoma - Non-Hodgkin: Statistics*, <<https://www.cancer.net/cancer-types/lymphoma-non-hodgkin/statistics>> (

135 cancer.net. *Lymphoma - Hodgkin: Statistics*, <<https://www.cancer.net/cancer-types/lymphoma-hodgkin/statistics>> (

136 cancer.org. *Survival Rates for Breast Cancer*, <<https://www.cancer.org/cancer/breast-cancer/understanding-a-breast-cancer-diagnosis/breast-cancer-survival-rates.html>> (

137 cancer.org. *Survival Rates for Melanoma Skin Cancer*, <<https://www.cancer.org/cancer/melanoma-skin-cancer/detection-diagnosis-staging/survival-rates-for-melanoma-skin-cancer-by-stage.html>> (

138 cancer.org. *Survival Rates for Prostate Cancer*, <<https://www.cancer.org/cancer/prostate-cancer/detection-diagnosis-staging/survival-rates.html>> (

139 Rees, J. R. *et al.* Survival after squamous cell and basal cell carcinoma of the skin: A retrospective cohort analysis. *INTERNATIONAL JOURNAL OF CANCER* **137**, 878-884, doi:10.1002/ijc.29436 (2015).

140 cancer.org. *Survival Rates for Soft Tissue Sarcoma*, <<https://www.cancer.org/cancer/soft-tissue-sarcoma/detection-diagnosis-staging/survival-rates.html>> (

141 cancer.org. *Survival Rates for Bladder Cancer*, <<https://www.cancer.org/cancer/bladder-cancer/detection-diagnosis-staging/survival-rates.html>> (

142 cancer.org. *Survival Rates for Mesothelioma*, <<https://www.cancer.org/cancer/malignant-mesothelioma/detection-diagnosis-staging/survival-statistics.html>> (

143 Kim, D. W. *et al.* Pilot study of intratumoral (IT) cryoablation (cryo) in combination with systemic checkpoint blockade in patients with metastatic melanoma (MM). *Journal for immunotherapy of cancer* **3**, P137-P137, doi:10.1186/2051-1426-3-S2-P137 (2015).

144 Mooradian, M. *et al.* The use of cryoablation to overcome resistance to PD-1 blockade in unresectable melanoma. *Journal of clinical oncology* **39**, 9538-9538, doi:10.1200/JCO.2021.39.15_suppl.9538 (2021).

145 Comen, E. A. *et al.* Preoperative checkpoint inhibition (CPI) and cryoablation (Cryo) in women with early-stage breast cancer (ESBC). *Journal of clinical oncology* **37**, 592-592, doi:10.1200/JCO.2019.37.15_suppl.592 (2019).

146 Wetzel, R. *et al.* A pilot study of the combination of checkpoint inhibition with ablation in subjects with biliary tract cancer. *Journal of clinical oncology* **39**, e16150-e16150, doi:10.1200/JCO.2021.39.15_suppl.e16150 (2021).

147 Campbell, M. T. *et al.* Pilot study of Tremelimumab with and without cryoablation in patients with metastatic renal cell carcinoma. *Nature communications* **12**, 6375-6375, doi:10.1038/s41467-021-26415-4 (2021).

148 Ross, A. E. *et al.* A pilot trial of pembrolizumab plus prostatic cryotherapy for men with newly diagnosed oligometastatic hormone-sensitive prostate cancer. *PROSTATE CANCER AND PROSTATIC DISEASES* **23**, 184-193, doi:10.1038/s41391-019-0176-8 (2020).

149 Thomsen, L. C. V. *et al.* A prospective phase I trial of dendritic cell-based cryoimmunotherapy in metastatic castration-resistant prostate cancer. *Journal of clinical oncology* **38**, 3029-3029, doi:10.1200/JCO.2020.38.15_suppl.3029 (2020).

150 McArthur, H. L. *et al.* A pilot study of preoperative single-dose ipilimumab and/or cryoablation in women with early-stage breast cancer with comprehensive immune profiling. *Clinical cancer research* **22**, 5729-5737, doi:10.1158/1078-0432.CCR-16-0190 (2016).

151 Lyu, N. *et al.* Ablation Reboots the Response in Advanced Hepatocellular Carcinoma With Stable or Atypical Response During PD-1 Therapy: A Proof-of-Concept Study. *Frontiers in oncology* **10**, doi:10.3389/fonc.2020.580241 (2020).

152 Shi, L. *et al.* 949P Thermal ablation plus toripalimab in patients with advanced hepatocellular carcinoma: Phase I results from a multicenter, open-label, controlled phase I/II trial (IR11330). *Annals of oncology* **32**, S826-S826, doi:10.1016/j.annonc.2021.08.169 (2021).

153 Monge B, M. C. *et al.* Long-term survival of combined ablation therapy and tremelimumab with or without durvalumab in advanced hepatocellular carcinoma. *Journal of clinical oncology* **38**, e16689-e16689, doi:10.1200/JCO.2020.38.15_suppl.e16689 (2020).

154 Segal, N. H. *et al.* Non-randomized phase II study to assess the efficacy of pembrolizumab (Pem) plus radiotherapy (RT) or ablation in mismatch repair proficient (pMMR) metastatic colorectal cancer (mCRC) patients. *Journal of clinical oncology* **34**, 3539-3539, doi:10.1200/JCO.2016.34.15_suppl.3539 (2016).

155 Xie, C. *et al.* Tremelimumab in Combination With Microwave Ablation in Patients With Refractory Biliary Tract Cancer. *Hepatology* **69**, 2048-2060, doi:10.1002/hep.30482 (2019).

156 Agdashian, D. *et al.* The effect of anti-CTLA4 treatment on peripheral and intra-tumoral T cells in patients with hepatocellular carcinoma. *CANCER IMMUNOLOGY IMMUNOTHERAPY* **68**, 599-608, doi:10.1007/s00262-019-02299-8 (2019).

157 Duffy, A. G. *et al.* Tremelimumab in combination with ablation in patients with advanced hepatocellular carcinoma. *JOURNAL OF HEPATOLOGY* **66**, 545-551, doi:10.1016/j.jhep.2016.10.029 (2017).

158 Qiao, G. *et al.* Immune correlates of clinical benefit in a phase I study of hyperthermia with adoptive T cell immunotherapy in patients with solid tumors. *International journal of hyperthermia* **36**, 74-82, doi:10.1080/02656736.2019.1647350 (2019).

159 OâNeill, C. *et al.* A phase 1b trial of concurrent immunotherapy and irreversible electroporation in the treatment of locally advanced pancreatic adenocarcinoma. *Surgery* **168**, 610-616, doi:10.1016/j.surg.2020.04.057 (2020).
